# Does time ever fly or slow down? The difficult interpretation of psychophysical data on time perception

**DOI:** 10.3389/fnhum.2014.00415

**Published:** 2014-06-10

**Authors:** Miguel A. García-Pérez

**Affiliations:** Departamento de Metodología, Facultad de Psicología, Universidad ComplutenseMadrid, Spain

**Keywords:** perception of duration, psychophysical methods, psychophysical function, psychometric function, probabilistic models

## Abstract

Time perception is studied with subjective or semi-objective psychophysical methods. With subjective methods, observers provide quantitative estimates of duration and data depict the *psychophysical function* relating subjective duration to objective duration. With semi-objective methods, observers provide categorical or comparative judgments of duration and data depict the *psychometric function* relating the probability of a certain judgment to objective duration. Both approaches are used to study whether subjective and objective time run at the same pace or whether time flies or slows down under certain conditions. We analyze theoretical aspects affecting the interpretation of data gathered with the most widely used semi-objective methods, including single-presentation and paired-comparison methods. For this purpose, a formal model of psychophysical performance is used in which subjective duration is represented via a psychophysical function and the scalar property. This provides the timing component of the model, which is invariant across methods. A decisional component that varies across methods reflects how observers use subjective durations to make judgments and give the responses requested under each method. Application of the model shows that psychometric functions in single-presentation methods are uninterpretable because the various influences on observed performance are inextricably confounded in the data. In contrast, data gathered with paired-comparison methods permit separating out those influences. Prevalent approaches to fitting psychometric functions to data are also discussed and shown to be inconsistent with widely accepted principles of time perception, implicitly assuming instead that subjective time equals objective time and that observed differences across conditions do not reflect differences in perceived duration but criterion shifts. These analyses prompt evidence-based recommendations for best methodological practice in studies on time perception.

“There is nothing so practical as a good theory”

Lewin (1951, p. 169)

“There is nothing so theoretical as a good method”

Greenwald (2012)

## Introduction

Time is crucial in our lives. We do not have a sense organ for time, but even infants 3–4 months old show some ability to discriminate short durations of different lengths (Provasi et al., 2011; Gava et al., 2012). During childhood and adolescence we develop a fine-grained perception of time surely based on our daily experience with objective time. Finely-tuned time perception seems to arise after higher-level cognitive processes are sufficiently developed (Block et al., 1999; Droit-Volet, 2013) and given explicit experience with objective time. It is nevertheless unclear whether our ability to represent and quantify time stems from a timing mechanism (an “internal clock”) that keeps track of time and can be read like a watch or, rather, only reflects our learning to translate experienced intervals into magnitudes expressed in the physical units of time that we got accustomed to. In the former case, empirical differences between subjective and objective time would be caused by an acceleration or deceleration of the internal clock, which thus gives an inexact reading (i.e., the internal clock is fast or slow); in the latter, they would reflect a subjective lengthening or shortening of duration, which nevertheless gets properly quantified afterwards. Figuring out which of these processes is taking place seems impossible because the process is unobservable and any observable outcome is compatible with these two and maybe also other accounts (Block, 1990; Grondin, 2010). Mechanistic accounts of timing processes have the status of metaphors (Wackermann, 2011), but difficulties to unravel those processes does not reduce our interest in investigating the phenomenon of time perception and the factors that affect it.

Time perception studies range from descriptions of the limits of our ability to judge and discriminate elapsed time or time differences, through the study of subject variables or stimulus conditions that affect such judgments, to assessments of distorted time perception in patients with psychiatric or neurological disorders. Detailed reviews have been published that describe the state and outcomes of this research in several areas (e.g., Grondin, 2010; Spence and Parise, 2010; Vroomen and Keetels, 2010; Allman and Meck, 2012; Chen and Vroomen, 2013; Allman et al., 2014) and this paper will not provide yet another review of this type. Our focus is instead on the methods used to gather data on the relation between subjective and objective time and on the conflicting or puzzling results that use of alternative (and presumably interchangeable) methods sometimes provides. Our main goal is to analyze the assumptions underlying these methods and to derive implications on the interpretation of data gathered with them. For this purpose, widely accepted principles of time perception will be built into a model of performance in psychophysical tasks in order to analyze the underpinnings, implications, and shortcomings of the various methods. To define our context, section Experimental Methods Used in Studies on Time Perception gives a brief overview of the classes of subjective and semi-objective methods used in studies on time perception. Section A Unified Model of Performance across Semi-Objective Psychophysical Tasks presents a model of performance in semi-objective psychophysical tasks that includes widely accepted components. Application of the model to different tasks in sections Single-Presentation Methods and Paired-Comparison Methods reveals how they can render conflicting results when time perception as implemented in the model is invariant across tasks. These results and their implications for best research practices are discussed in section General Discussion and Evidence-Based Recommendations.

## Experimental methods used in studies on time perception

**Figure 1:**
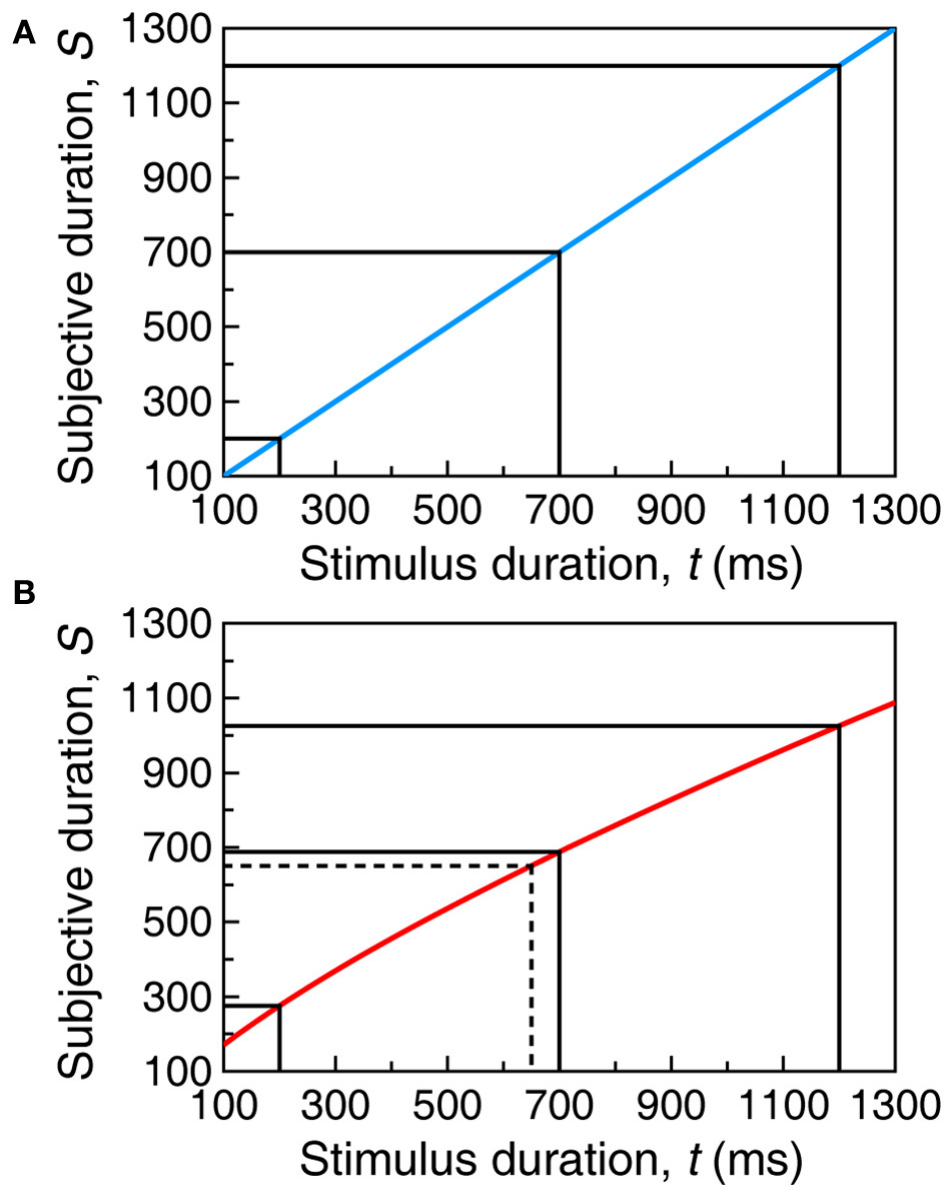
**Sample psychophysical functions described by Equation (1).** The psychophysical function describes the mapping of objective time (in physical units, e.g., ms) onto subjective time (in arbitrary units). **(A)** With α = 1, β = 1, and τ = 0, μ is the identity function by which subjective duration equals objective duration. **(B)** With α = 5, β = 0.75, and τ = −10, μ is a concave function by which durations shorter than *t* = 654 ms are subjectively perceived longer than they are whereas increasingly longer durations are progressively compressed. Solid black lines illustrate the mapping for sample durations *t* = 200 ms, *t* = 1200 ms, and their midpoint at *t* = 700 ms. Dashed lines in the bottom panel illustrate that, due to the non-linear μ, the midpoint between μ(200) and μ (1200) on the vertical axis does not correspond to the midpoint between *t* = 200 ms and *t* = 1200 ms on the horizontal axis.

Research on time perception comprises two major types of study (Grondin, 2010): retrospective and prospective. *Retrospective studies* assess remembered time by asking observers to quantify the time elapsed while they had been engaged in a task performed without knowledge that their time estimates would be eventually assessed (e.g., Kellaris and Kent, 1992; Friedman and Kemp, 1998; Campbell and Bryant, 2007; Arstila, 2012; Misuraca and Teuscher, 2013; Dong and Wyer, 2014). In contrast, *prospective studies* assess immediately experienced time through psychophysical tasks that can be categorized as subjective or semi-objective. In subjective methods, observers report the perceived duration of the stimulus presented in each trial, whether through the *verbal estimation task* (requesting a numerical estimate of presentation duration), the *temporal reproduction task* (asking observers to reproduce a duration of the same length), or the *temporal production task* (asking observers to produce a duration lasting the amount of time indicated verbally). The ultimate goal of subjective methods is to estimate the *psychophysical function* expressing the functional relation of subjective to objective time (see Figure 1), a historical endeavor of classical psychophysics (Eisler, 1976; Allan, 1983).

**Figure 2:**
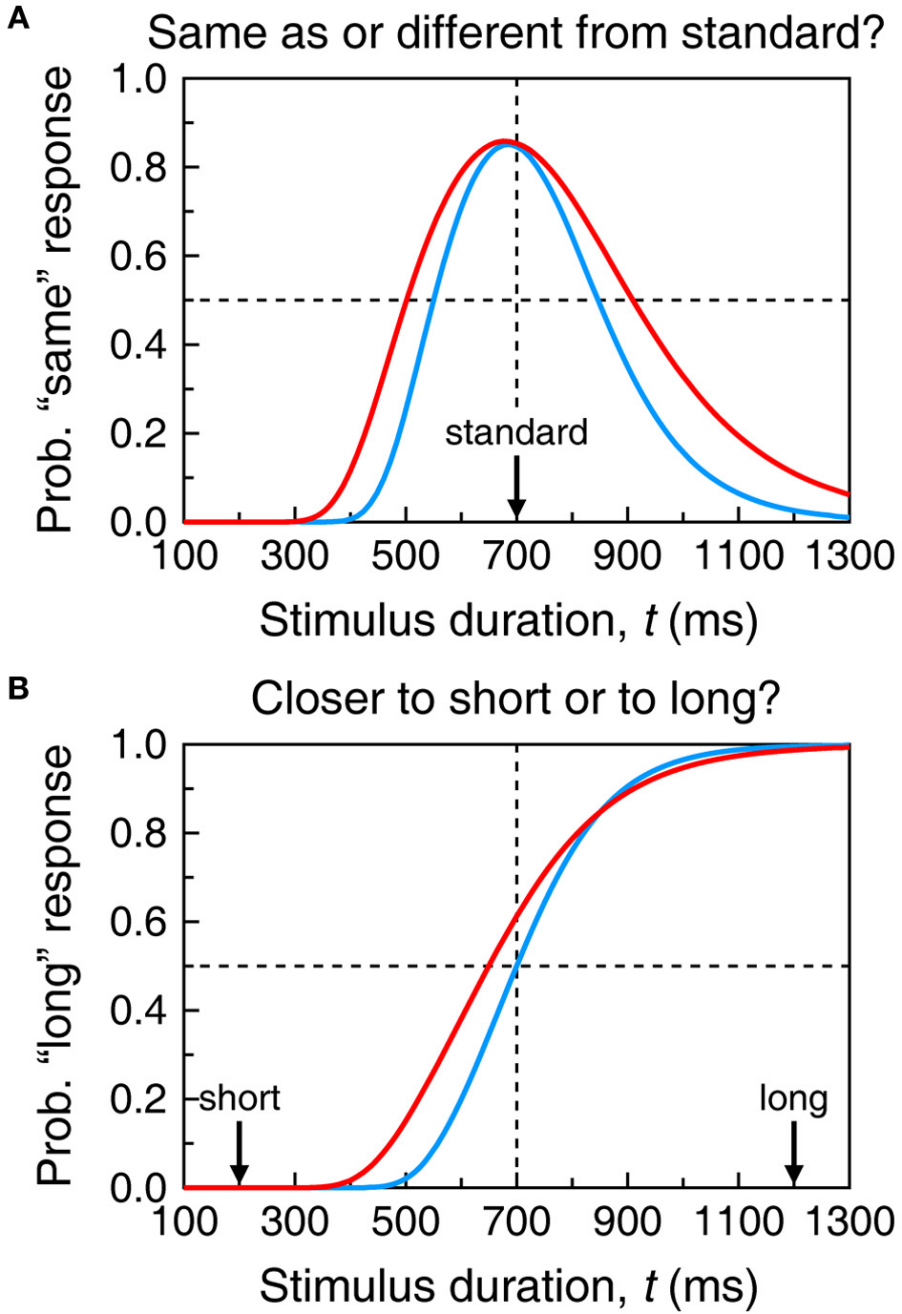
**Sample psychometric functions in the temporal generalization task (A) and the temporal bisection task (B).** Each function in each panel results from the psychophysical function plotted with the same color in Figure 1, with additional assumptions coming from the models described in sections A Unified Model of Performance across Semi-Objective Psychophysical Tasks and Single-Presentation Methods. Specifically, in the top panel, δ = 150 whereas in the bottom panel, δ = 70 and ξ = 0.5. In both cases γ = 0.15. The red curve in **(A)** is the same psychometric function plotted in Figure 4A; the red curve in **(B)** is the same psychometric function plotted with continuous black trace in Figure 5A.

Semi-objective methods also involve the display of stimuli whose presentation duration varies across trials but observers are not requested to produce quantitative estimates. Instead, they are asked for categorical or comparative judgments. Semi-objective tasks include single-presentation methods and paired-comparison methods. In the former, each trial presents a single stimulus and a categorical response is requested; in the latter, two stimuli are presented in each trial and a comparison is requested. (Multiple-comparison methods involving three or more stimuli per trial will not be discussed here.) Single-presentation methods include the *bisection task* (asking observers to report whether the currently displayed duration is closer to a short or to a long exemplar repeatedly displayed in a preceding training phase) and the *temporal generalization task* (asking observers to report whether or not the currently displayed duration is the same as an exemplar duration also repeatedly displayed in a preceding training phase). Paired-comparison methods include the *two-alternative forced-choice (2AFC)* or *comparative task* (asking observers to indicate which of the two stimuli in each trial had, say, a longer duration) and the *equality* or *same–different task* (asking observers to indicate whether or not the two stimuli had the same duration), although many other variants exist. Almost invariably, semi-objective methods are used to estimate the *psychometric function* describing how the probability of some response varies with duration (see Figure 2). Landmark points on the psychometric function are subsequently extracted to characterize aspects of time perception, including the bisection point (BP, the 50% point on the psychometric function in a bisection task), the point of subjective equality (PSE, the location of the peak of the psychometric function in the temporal generalization task or the 50% point on the psychometric function in the 2AFC task), or the difference limen (DL, a measure of the spread of a psychometric function). Also often computed is the Weber ratio (WR) defined as either DL/BP or DL/PSE. Superficially, psychometric functions estimated with semi-objective methods offer an account that differs from that provided by psychophysical functions estimated with subjective methods. Yet, the psychometric function embeds the psychophysical function, as will be seen later.

Prospective studies typically include several conditions to investigate differences in subjective time across experimental manipulations (Ulbrich et al., 2007; Wearden et al., 2010; Ogden, 2013) or subject variables (Carlson and Feinberg, 1970; Eisler and Eisler, 1994; Glicksohn and Hadad, 2012). Differences in time perception could naturally be expected to occur as a result of these factors. Our theoretical analyses will assess how the use of alternative semi-objective tasks and the way in which data are analyzed can speak about these differences.

## A unified model of performance across semi-objective psychophysical tasks

This section presents a unified model of performance in all the semi-objective psychophysical tasks used to investigate time perception. Specific models have been proposed for individual tasks, but they are not always applicable to other tasks and, thus, they offer a fragmentary view of time perception. The model used for our purpose here extends the signal detection theory (SDT) model of Gibbon (1981), which was indeed the basis for most models of performance in semi-objective tasks. The model includes a timing component and a decisional component determining how observers use the outcome of the timing component to make a judgment and give a response.

For the timing component, the model assumes that objective time is internally represented as described by the psychophysical function μ, irrespective of the mechanism by which this representation is obtained. The psychophysical function μ reflects a mapping of objective onto subjective time that can be measured with subjective methods. This does not imply that the psychophysical function estimated with those methods for some particular stimulus should exactly govern the judgments expressed by observers in semi-objective tasks with the same stimulus. Psychophysical functions vary with the subjective method used to estimate them (Carlson and Feinberg, 1970; Angrilli et al., 1997; Gil and Droit-Volet, 2011), but also with instructions (Rattat and Droit-Volet, 2012) or with the interface used to collect responses (Mioni et al., 2014). Yet, judgments reported in semi-objective tasks must arise from a representation of time analogous to that subserving performance in subjective tasks. Extensive research has shown that the psychophysical function for duration is well-approximated by the three-parameter power function

(1) $$\mu (t)=\alpha \text{​}{\left(t\;-\;\tau \right)}^{\beta },$$

with an exponent β close to unity and a shift τ close to zero. Parameter values vary across stimulus types and experimental conditions (Marks and Stevens, 1968; Fagot, 1975; Eisler, 1976; Dawson and Miller, 1978; Allan, 1983) and one must consider a family μ_*i*_, in which the subscript (also in the parameters) denotes condition. Within a condition, subjective duration exceeds objective duration within the range of *t* for which μ*_i_(t)* > *t* whereas subjective duration is shorter than objective duration wherever μ*_i_(t)* <*t*. Figure 1 showed two psychophysical functions described by Equation (1). If μ(*t*) = *t* (Figure 1A), subjective and objective time run identically; if β ≠ 1 (Figure 1B), subjective time runs faster or slower than real time (see Gibbon, 1986, his Figure 1). Across conditions, μ*_i_(t)* ≠ μ*_j_(t)* implies that time runs (relatively) faster in one condition than in the other.

The parameters of μ are estimated from the durations reported by observers across repeated presentations of a set of objective durations. The fitted function thus reflects the average subjective duration of a stimulus of duration *t*. Scalar expectancy theory derived from studies with non-human animals (Church and Deluty, 1977; Gibbon, 1977; Church and Gibbon, 1982) posits that the standard deviation of subjective duration is proportional to the average subjective duration at *t*, namely,

(2) σ(t)=γμ(t).

**Figure 3:**
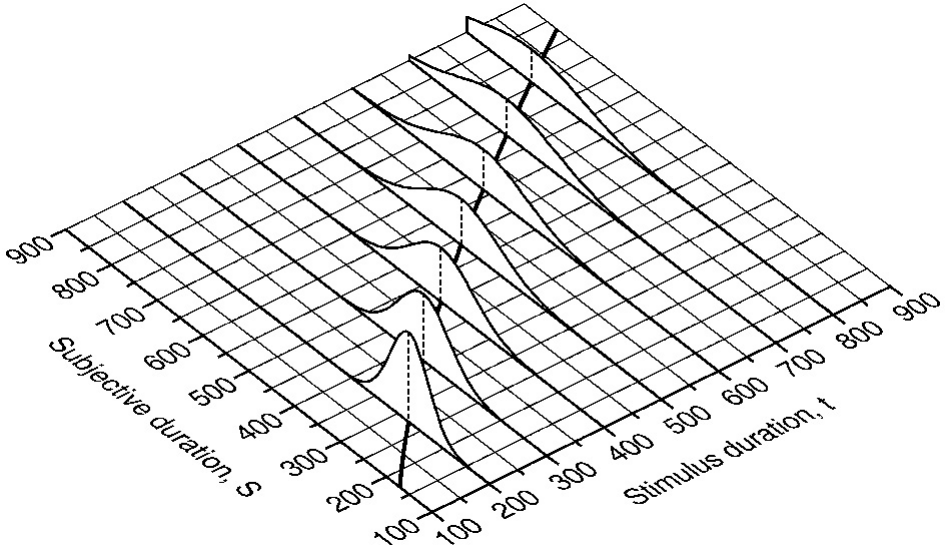
**Distributions of subjective duration at selected values of objective duration.** The mean of each distribution is given by Equation (1) with the same parameter values used in the bottom panel of Figure 1 (the partly-occluded thick curve on the plane surface shows this psychophysical function). The distributions obey the scalar property in Equation (2) with γ = 0.15.

This is known as the *scalar variance* assumption or the *scalar property*. A family of functions σ_*i*_ must also be considered across conditions. With scalar variance, the coefficient of variation σ*_i_(t)*/μ*_i_(t)* of the distribution of subjective durations equals γ_*i*_ at all *t*. Scalar variance holds only approximately in human timing although the standard deviation certainly increases with *t* (Wearden, 1991b; Lewis and Miall, 2009). In any case, the subjective duration *S* of a stimulus of duration *t* under condition *i* can be regarded as a random variable with mean μ*_i_(t)* and standard deviation σ*_i_(t)* and, without loss of generality, *S* is assumed to be normally distributed (Figure 3). This provides the output of the timing component in the model.

This characterization implies that the subjective duration elicited by presentation of a stimulus of duration *t* is a random value sampled from the applicable distribution, regardless of the psychophysical task or the occasion that motivated the presentation of such stimulus. In semi-objective psychophysical tasks, observers are assumed to make a decision and respond according to the values drawn for each of the stimuli presented in each trial. Modeling performance on these tasks thus calls for a decision rule specifying how observers use the current sample (or samples) of subjective duration to make a judgment and give a response. This decisional component must vary across tasks but its elements must be consistent in the sense that the decision rule for some task cannot imply aspects or processes that are explicitly regarded as inexistent or impossible under alternative tasks. This is a reasonable demand on consideration that trials from different tasks can be interwoven in a session, with the response requested on each trial withheld until after stimulus presentation. In such conditions, duration(s) must be internally represented before observers know which decision rule must be used to give a response. Empirical evidence shows that the operation of the stimulus-dependent component precedes and is unaffected by the task-dependent decisional component (Schneider and Komlos, 2008; García-Pérez and Alcalá-Quintana, 2012; García-Pérez and Peli, 2014). On the same grounds, one must assume that μ_*i*_ and σ_*i*_ do not vary across tasks when stimuli and conditions are invariant.

Sections Single-Presentation Methods and Paired-Comparison Methods describe the model (timing output and decision rule) describing performance on the most common semi-objective psychophysical tasks, also discussing other assumptions needed to interpret the data.

## Single-presentation methods

In single-presentation methods, observers are shown a single stimulus on each trial for them to report a categorical judgment. Making this judgment nevertheless requires that the internal representation of the current stimulus is judged relative to what has sometimes been called an *internal standard*. The two methods described next differ as to how the internal standard is instated and what type of categorical judgment is requested.

### The temporal generalization task

The temporal generalization task consists of a training phase and a test phase. In the training phase, observers are repeatedly shown instances of an exemplar duration *t*_st_ designated as the standard. The test phase comprises a series of trials each of which displays a duration *t* around *t*_st_ and asks observers to indicate whether that duration was the same as *t*_st_. A plot of the proportion of “same” responses as a function of test duration describes the empirical psychometric function.

When both phases use the same stimulus and conditions are identical, subjective duration must be governed by the same μ and σ in both phases. Hence, the data are expected to reveal that the test duration at which “same” responses are maximally prevalent is *t* = *t*_st_. But stimuli or conditions may differ across phases: The training phase may use a neutral stimulus such as an oval while the test phase uses emotional stimuli such as angry faces or taboo words. In such case, μ and σ will differ across phases if subjective time runs differently in each condition. Let subscripts “s” and “t” respectively denote the functions that apply to the standard and to the test. Then, one would expect the data to disclose the test duration whose subjective duration equals the subjective duration of the standard. Formally, this is the value *t*_PSE_ at which μ_t_(*t*_PSE_) = μ_s_(*t*_st_).

Fitting model-based psychometric functions to the data is useful for these purposes. Formal models from which theoretical psychometric functions for the temporal generalization task can be derived were first proposed by Church and Gibbon (1982) and Wearden (1992). The model described next differs from these in minor respects discussed in section Differences with Previous Models.

#### Model and assumptions

The training phase helps observers to set an anchor point (internal standard) from the sample of subjective durations elicited by repeated presentation of the standard. The anchor is presumably placed at *S*_st_ = μ_s_(*t*_st_) and kept invariant. In the test phase, observers compare the random subjective duration *S* elicited by the current test with *S*_st_ and respond according to the magnitude of |*S* − *S*_st_|. Observers are assumed to have a limited resolution to tell small differences from zero, for otherwise they would always respond “different.” Under these assumptions, the decision rule states that observers respond “same” when |*S* − *S*_st_| ≤ δ and “different” when |*S* − *S*_st_| > δ, where δ is the *resolution limit* and the interval from *S*_st_ − δ to *S*_st_ + δ is the *indifference region*.

**Figure 4:**
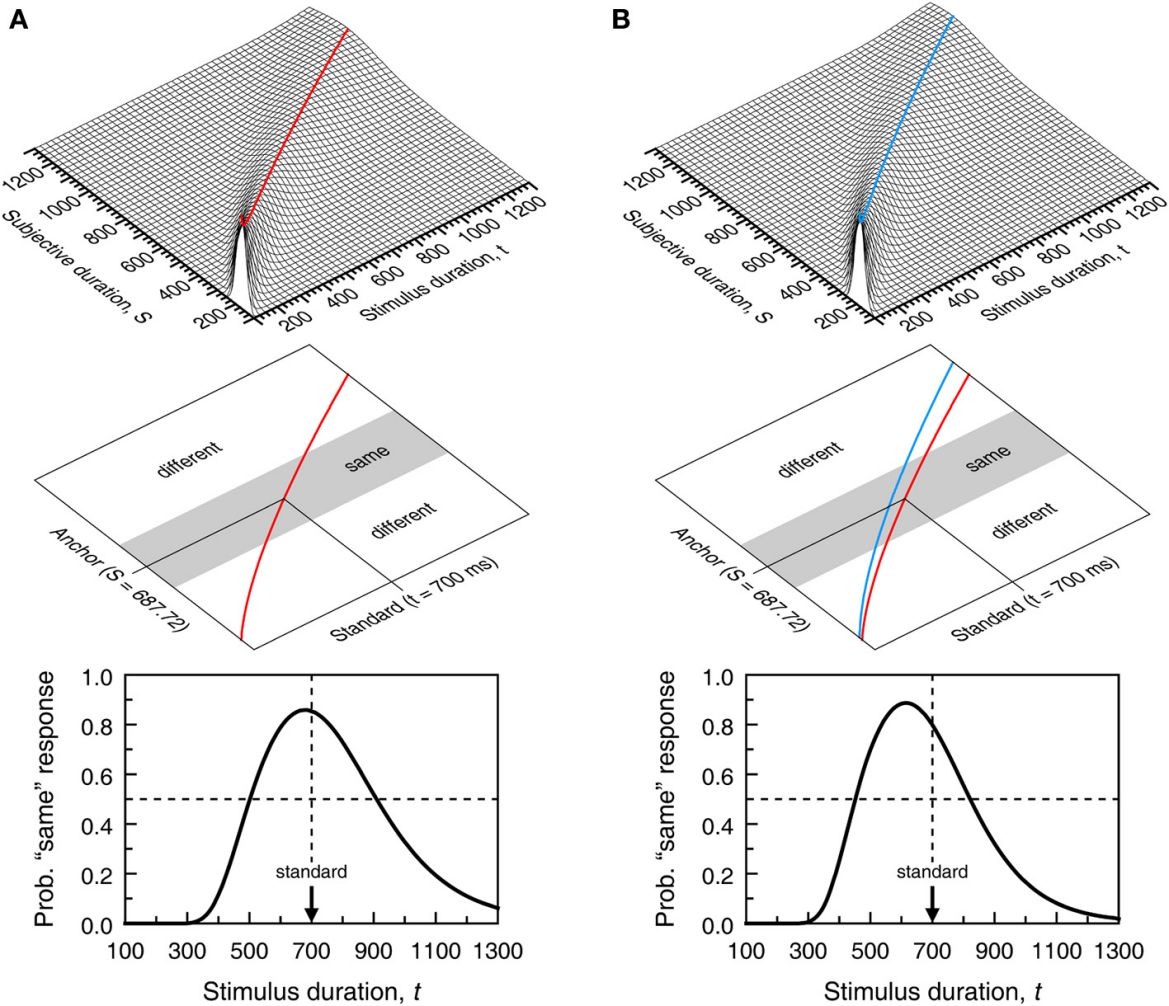
**Model-based psychometric functions for the temporal generalization task.** In **(A)**, perception of duration is governed by the same psychophysical function for training and test stimuli; in **(B)**, they are governed by different psychophysical functions. The top panel shows a continuous version of the surface for which cross-sectional plots at selected *t* were shown in Figure 3. The surface in **(A)** has the same parameters as in Figure 3 and governs perception of duration for the training stimulus in both columns and also for the test stimulus in **(A)**; the surface in **(B)** only differs in that α = 5.4 instead and is assumed to govern perception of duration of the test stimulus in **(B)**. The middle panel shows the partition of decision space into a central gray region of subjective durations that result in “same” responses and two outer white regions of subjective durations that result in “different” responses. The central gray region spans δ = 150 units on either side of the anchor point. The bottom panel shows the resultant psychometric function in Equation (3). The ordinate at each value of *t* equals the area under the cross-section at *t* of the surface in the top panel within the region that results in “same” responses.

The mathematical form of the psychometric function for “same” responses is easily derived from these assumptions. Given that *S*_st_ = μ (*t*_st_) is assumed constant and that *S* in the test phase is normally distributed with mean μ_t_*(t)* and standard deviation σ_t_(*t*), the probability Ψ_same_ of a “same” response varies with test duration *t* as

(3) $$\begin{array}{l} \Psi_{\text{same}}(t)=\text{Prob}\left(\left|S-S_{\text{st}}\right|\leq \delta \right)\\ \;=\text{Prob}\left(S_{\text{st}}-\delta \leq S\leq S_{\text{st}}+\delta \right)\\ \;=\Phi \text{​}\left(\frac{S_{\text{st}}+\delta -\mu_{\text{t}}(t)}{\sigma_{\text{t}}(t)}\right)-\Phi \text{​}\left(\frac{S_{\text{st}}-\delta -\mu_{\text{t}}(t)}{\sigma_{\text{t}}(t)}\right),\;\;\; \end{array}$$

where Φ is the unit-normal cumulative distribution function. The psychophysical function μ is thus embedded in the psychometric function, as is the scalar property. Figure 4A shows the psychometric function when μ_s_ = μ_t_ ≡ μ and σ_s_ = σ_t_ ≡ σ (see the legend for parameter values) so that Equation (3) becomes

(4) $$\Psi_{\text{same}}(t)=\Phi \text{​}\left(\frac{S_{\text{st}}+\delta -\mu (t)}{\sigma (t)}\right)-\Phi \text{​}\left(\frac{S_{\text{st}}-\delta -\mu (t)}{\sigma (t)}\right).$$

Even in these conditions, Ψ_same_ does not peak at the standard duration because *t* = *t*_st_ maximizes Equation (4) only when σ(*t*) is a constant function independent of *t*. When σ(*t*) obeys the scalar property, Equation (4) peaks at $t=\frac{\sqrt{1+4\gamma^{2}}-1}{2\gamma^{2}}S_{\text{st}}$. The right-hand side of this expression evaluates to 0.99*S*_st_ when γ = 0.1 and to 0.92*S*_st_ when γ = 0.3, but the spacing used in empirical studies (typically 100–200 ms) is too coarse to reveal this shift. If differences in stimuli or conditions across training and test phases affect subjective time, Ψ_same_ is further shifted because the peak of Equation (3) is further away from *t* = *t*_st_ when μ_s_ ≠ μ_t_ (see Figure 4B). Note also that differences between σ_s_ and σ_t_ are inconsequential, as σ_s_ plays no role in Ψ_same_. The gradients on either side of Ψ_same_ are determined by how σ_t_ varies with *t*. With scalar variance, Ψ_same_ is positively skewed. The skew is further emphasized when μ_t_ is a concave function (β_*t*_ < 1 in Equation 1) and reduced or even reversed when μ_*t*_ is convex (compare the two curves in Figure 2A).

Because Ψ_same_ does not peak at *t* = *t*_st_ even when μ_s_ = μ_t_, observed shifts of Ψ_same_ away from *t*_st_ cannot be interpreted as evidence that μ_s_ ≠ μ_t_ and, thus, of differences in subjective time in the conditions of the test phase relative to the training phase. Other difficulties in the interpretation of data from the temporal generalization task will be discussed in section Summary and Discussion of Single-Presentation Methods.

#### Differences with previous models

Wearden (1992) proposed three variants of the model of Church and Gibbon (1982), which are discussed next in our notation. All variants share two characteristics: (1) subjective duration is assumed accurate on average so that the psychophysical function is μ (*t*) = *t* in all cases and (2) the internal standard is not regarded as a fixed value but as a random variable with mean *t*_st_. Also, the resolution parameter (called threshold by Wearden) is fixed at δ in some variants but regarded as random with mean δ in others. All three variants use a decision rule analogous to that in section Model and Assumptions and they differ as to the assumed variances of subjective duration, internal standard, and threshold (when random).

The *modified-Church-and-Gibbon* (MCG) model assumes σ(*t*) = 0. Thus, subjective duration is not a random variable and, given μ(*t*) = *t*, it is identical to objective duration. This model places the scalar property at the internal standard (drawn in each trial from the memory representation of the standard) whereas the threshold is regarded as a random variable with fixed variance. The MCG model is structurally equivalent to our model because the distribution of |*X* − *Y*| is the same regardless of which of *X* or *Y* is the random variable and which is the constant and also because the variability of δ can be formally transferred to the internal standard. But this model presents an empirical difficulty: If subjective duration equals objective duration (a consequence of assuming μ(*t*) = *t* and σ(*t*) = 0), observers would be perfectly accurate in paired-comparison tasks asking them to judge the relative durations of two stimuli displayed in each trial (see section Paired-Comparison Methods).

The *fixed-threshold* model removes the variability of δ while leaving other assumptions of the MCG model intact. This model is also formally equivalent to our model and to the MCG model, and results reported by Wearden (1992; see his Table 1) reveal that the estimated variability of the internal standard increases under this model to capture the variability attributed to threshold under the MCG model. Finally, the *timing-variability* model assumes scalar variance for subjective duration in place of σ(*t*) = 0, also assuming scalar variance with the same γ for the internal standard and a threshold randomly drawn in each trial from a distribution with fixed variance. Structurally, this model is not equivalent to the others because it involves a ratio of independent normal random variables, whose distribution is not normal (Simon, 2002, formula 7.7). The model nevertheless produces nearly identical psychometric functions and is also functionally equivalent to the previous two and to our model, although scalar variance affects two random variables here and must result in smaller estimates of γ to account for the same data (see Table 1 in Wearden, 1992).

Because all the models of Wearden (1992) use μ(*t*) = *t*, they explicitly assume that subjective and objective time run identically and, hence, the models are incompatible with the notion that subjective time may run at a different pace, or with an interest in assessing what that pace may be and how it varies across conditions. Fitting these models to empirical data enforces the assumption of veridical time perception and succeeding at that shows that temporal generalization data are compatible with the notion that subjective time is equivalent to objective time. This outcome is not to be taken as a proof that time perception is never distorted relative to objective time but as a manifestation of non-identifiability issues hampering the interpretation of data, whose discussion is deferred to section Summary and Discussion of Single-Presentation Methods.

### The temporal bisection task

The temporal bisection task also consists of a training phase and a test phase. In the training phase, observers are shown repeated instances of exemplar durations *t*_short_ and *t*_long_ designated short and long, respectively. The test phase comprises trials displaying a test duration *t* typically between *t*_short_ and *t*_long_. Observers are asked to judge whether the current test duration is closer to the short or to the long exemplars. A plot of the proportion of “long” responses at each test duration describes the empirical psychometric function and the 50% point on this function is taken to be the BP.

Performance is governed by common μ and σ if stimuli and conditions do not differ across phases. The BP might then be expected to lie at the midpoint between *t*_short_ and *t*_long_ only if μ is linear (Figure 1A and the blue curve in Figure 2B). With non-linear μ, the objective midpoint does not map onto the subjective midpoint (Figure 1B) and the BP would be expected to lie at the test duration associated with the subjective midpoint (red curve in Figure 2B). Note that the two cases in Figure 2B reflect an exquisite ability to bisect the subjective continuum; the different BPs simply reflect the form of μ. Bisection tasks are also used with different stimuli or conditions in the training and test phases so that μ_t_ may differ from μ_s_ and σ_t_ may differ from σ_s_ (using the same notation as before). In such cases, one expects the BP to identify the test duration that is subjectively midway between the subjective durations of the short and long standards.

Quite often, cumulative Gaussian or logistic functions are fitted to data to estimate the BP and the DL from location and slope parameters. Formal models from which suitable psychometric functions can be derived were first proposed by Gibbon (1981) and Wearden (1991a; see also Wearden and Ferrara, 1995). The model described next differs from these in some respects discussed in section Differences with Previous Models.

#### Model and assumptions

The training phase helps observers to set anchor points from the sample of subjective durations elicited by repeated presentation of short and long exemplars. In principle, the anchor points are assumed to be placed at *S*_s_ = μ_s_(*t*_short_) and *S*_l_ = μ_s_(*t*_long_) and also to be invariant. In the test phase, observers compare the subjective duration *S* of the current stimulus with *S*_s_ and *S*_l_ and respond according to which of |*S* − *S*_s_| or | *S* − *S*_l_| is the smallest. In principle, “long” responses are in order when |*S* − *S*_l_ | < |*S* − *S*_s_|, which simplifies to *S* > (*S*_s_ + *S*_l_)/2. For all purposes, this is as if observers set a single anchor at the subjective midpoint *S*_mp_ = (*S*_s_ + *S*_l_)/2. Assuming that observers use a point criterion and always classify *S* as closer to the short or the long exemplars is incompatible with assumptions in the model for temporal generalization: If observers can use a point criterion in the bisection task to decide whether *S* is above or below *S*_mp_, they should show the same capability in the temporal generalization task for deciding whether *S* is above or below *S*_st_, and thus they would always respond “different.” Observers surely have limited resolution also in the bisection task so that they respond “short” when *S* < *S*_mp_ − δ, respond “long” when *S* > *S*_mp_ + δ, and cannot tell when *S*_mp_ − δ ≤ *S* ≤ *S*_mp_ + δ, also involving a resolution limit and an indifference region. But, because observers are forced to respond “short” or “long,” they must use an extra criterion when they cannot tell. The model assumes that they respond “long” with probability ξ, reflecting their *response bias* and regardless of the criteria that render such outcome.

The psychometric function Ψ_long_ for “long” responses is easily derived from these assumptions. Since *S*_mp_ is assumed constant and *S* in the test phase is normally distributed with mean μ_t_(*t*) and standard deviation σ_t_(*t*), the probability of a “long” response varies with test duration *t* as

(5) $$\begin{array}{l} \;\Psi_{\text{long}}(t)=\text{Prob​}\left(S>S_{\text{mp}}+\delta \right)+\xi \text{Prob​}\left(S_{\text{mp}}-\delta \leq S\leq S_{\text{mp}}+\delta \right)\\ =\left[1-\Phi \text{​}\left(\frac{S_{\text{mp}}+\delta -\mu_{\text{t}}(t)}{\sigma_{\text{t}}(t)}\right)\right]+\xi [\Phi \text{​}\left(\frac{S_{\text{mp}}+\delta -\mu_{\text{t}}(t)}{\sigma_{\text{t}}(t)}\right)\\ \;-\Phi \text{​}\left(\frac{S_{\text{mp}}-\delta -\mu_{\text{t}}(t)}{\sigma_{\text{t}}(t)}\right)]\\ =1-\xi \Phi \text{​}\left(\frac{S_{\text{mp}}-\delta -\mu_{\text{t}}(t)}{\sigma_{\text{t}}(t)}\right)-\left(1-\xi \right)\text{​}\Phi \text{​}\left(\frac{S_{\text{mp}}+\delta -\mu_{\text{t}}(t)}{\sigma_{\text{t}}(t)}\right).\;\;\;\;\;\; \end{array}$$

**Figure 5:**
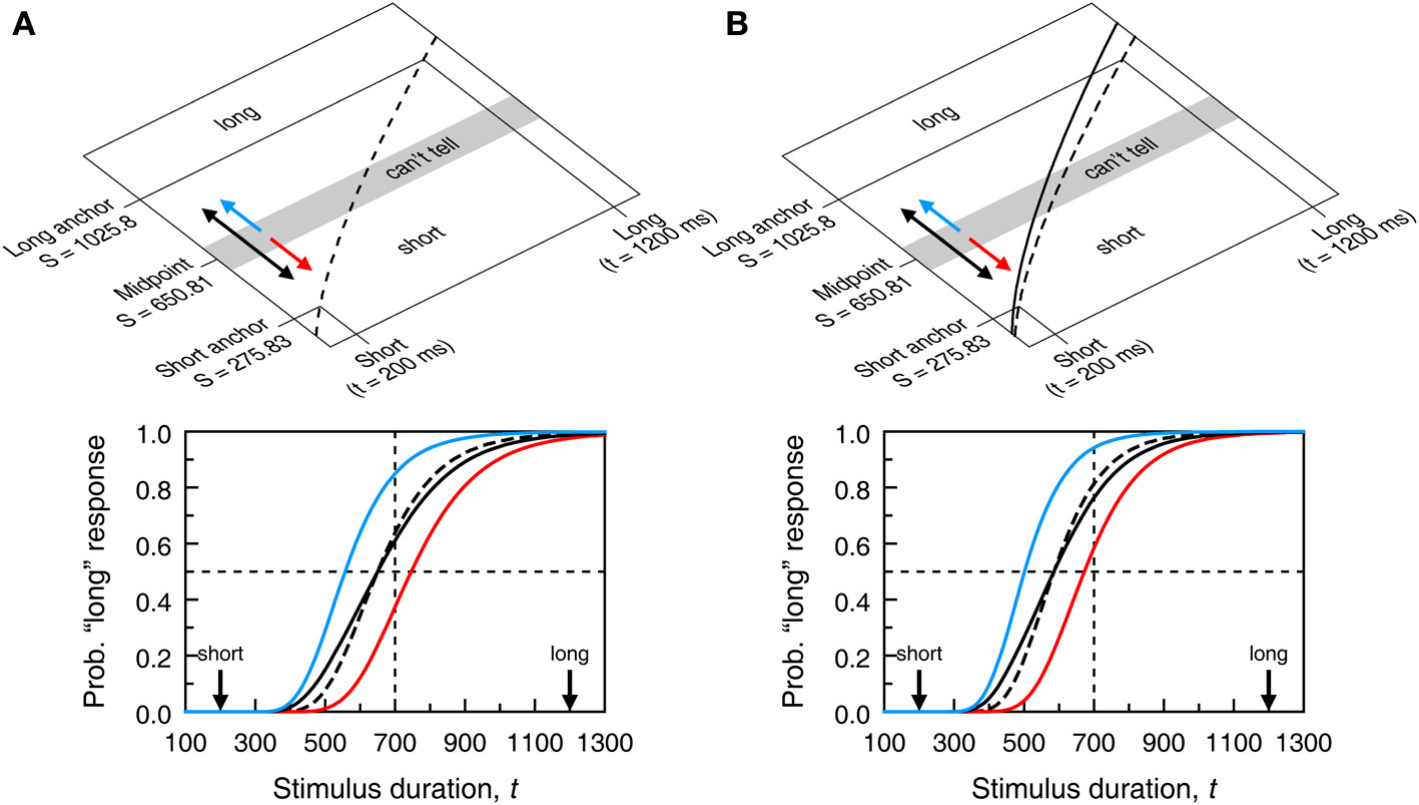
**Model-based psychometric functions for the temporal bisection task.** Panels **(A**,**B)** reflect the same scenarios as in Figure 4 regarding psychophysical functions (identified in the top panels here as dashed and solid curves instead of red and blue curves). The top panel shows a decision space analogously partitioned (now using δ = 70) but the regions now result in “short,” “I can't tell,” and “long” judgments although “I can't tell” judgments must still be reported as “short” or “long” responses. The bottom panel shows the psychometric functions (from Equation 5) that may result according to how observers respond when undecided. The blue curve arises when “I can't tell” judgments are always reported as “long” responses (blue arrow in the top panel; ξ = 1 in Equation 5); the red curve arises when “I can't tell” judgments are always reported as “short” responses (red arrow in the top panel; ξ = 0 in Equation 5); the black curve arises when “I can't tell” judgments are reported as “short” or “long” responses with equiprobability (double-headed black arrow in the top panel; ξ = 0.5 in Equation 5); the dashed curve arises if δ = 0 so that observers use a point criterion at the subjective midpoint and are never undecided.

Figure 5A shows sample psychometric functions when μ_s_ = μ_t_ = μ and σ_s_ = σ_t_ = σ for several values of the response bias parameter ξ. It is noteworthy that the location of the 50% point on Ψ_long_ varies greatly with ξ. In principle, only when μ_s_ and μ_t_ are linear does *t*_mp_ map onto *S*_mp_ (Figure 1). But, even when they are linear, Ψ_long_ has its 50% point at *t*_mp_ only when ξ = 0.5 (blue curve in Figure 2B). It is also noteworthy that the location and slope of Ψ_long_ are greatly affected by the irrelevant response bias and resolution parameters ξ and δ (compare with the dashed black curve for δ = 0 in the bottom panel of Figure 5A), undermining interpretation of the BP and the DL.

If differences in stimuli or conditions across training and test phases affect subjective time, Ψ_long_ is further shifted (Figure 5B) although given the influence of response bias, the 50% on Ψ_long_ carries no information that can be readily interpreted in terms of the pace of subjective time. As in temporal generalization, differences between σ_s_ and σ_t_ are inconsequential as σ_s_ plays no role in Ψ_long_. As resolution decreases (i.e., δ increases) with fixed ξ, Ψ_long_ becomes shallower (compare the dashed and solid black curves in the bottom panels of Figure 5). Finally, Ψ_long_ is not symmetric about its 50% point: Its left side rises more sharply than its right side levels off. This is due to the scalar property (for a discussion of this issue, see Killeen et al., 1997).

It is worth mentioning a mixed task in which a standard is used in the training phase (as in temporal generalization) and observers report whether the current test duration is longer or shorter than the standard (as in temporal bisection). In such case (Grondin and Rammsayer, 2003), observers do not need to build *S*_mp_ from *S*_s_ and *S*_l_ in the training phase but build *S*_st_ directly and use it in the test phase. The model psychometric function is still given by Equation (5) with *S*_st_ in place of *S*_mp_. Another variant of the bisection task is the “partition bisection” of Wearden and Ferrara (1995), in which the training phase is omitted and observers are simply asked to classify test stimuli as “short” or “long” using whichever criterion they wish. The psychometric function is again given by Equation (5), except that *S*_mp_ is a free parameter that captures the arbitrary criterion used by observers. In yet a further variant, observers receive feedback relative to the objective midpoint of the range of test durations (Grondin, 1998), which should help them to set a stable criterion.

#### Differences with previous models

The seminal model of Gibbon (1981) is analogous to the model just described except that he omitted the indifference region. His model thus arises by setting δ = 0 to revert to a point criterion. Gibbon analyzed versions of the model in which μ is non-linear and σ obeys the scalar property so that estimated model parameters speak of the pace at which subjective time runs. In addition, he considered the implications of decision rules involving point criteria other than *S*_mp_ = (*S*_s_ + *S*_l_)/2.

Wearden (1991a) adapted his fixed-threshold model of temporal generalization for application to bisection tasks, thus including the indifference region missing in Gibbon's (1981) model. In this model, observers draw random memories of the long and the short durations (both of which are accurate on average and have scalar variance) to compare them with the exactly perceived test duration (i.e., μ(*t*) = *t* and σ(*t*) = 0), responding “long” or “short” according to which distance is the smallest but provided that the difference of distances is beyond a fixed threshold (resolution limit). On trials in which the threshold is not exceeded, observers are undecided and always respond “long.” This model is formally equivalent to our model in Equation (5) with ξ = 1, μ_t_(*t*) = *t*, and σ_t_(*t*) reflecting instead the variability of the memory representations. Wearden and Ferrara (1995) later made two amendments to this model: Undecided observers respond “short” or “long” with equiprobability (ξ = 0.5) and the anchor *S*_mp_ is randomly drawn in each trial from a distribution whose mean equals the average of the set of test durations. This is the only random variable in the model but Wearden and Ferrara's (1995) writing is unclear about whether its standard deviation was fixed or increased with *t* so as to incorporate the scalar property.

By embedding the assumption that μ(*t*) = *t*, these models are unsuitable for assessing how subjective time runs compared to objective time. A 50% point found to be away from *t*_mp_ is implicitly attributed to response bias or to a criterion *S*_mp_ placed away from μ_s_(*t*_mp_) = *t*_mp_ (for an amendment of the model in this respect, see Wearden, 2004). Such decisional or response aspects are unrelated to time perception, which is regarded as accurate under these models. The same holds for the model of Killeen et al. (1997), which also assumes μ(*t*) = *t* and the scalar property but uses a logistic function as an approximation to Φ.

Kopec and Brody (2010) presented a model of an entirely different nature for the bisection task. This model is not considered here because it involves assumptions, processes, and decision rules that are specific to bisection tasks and cannot describe performance in any other task. For instance, applied to a temporal generalization task, the model posits that Ψ_same_ should have a symmetric Gaussian shape peaking at *t* = *t*_st_ and such that Ψ_same_(*t*_st_) = 1.

### Summary and discussion of single-presentation methods

Psychometric functions describing performance in single-presentation methods embed a representation of subjective duration (the functions μ and σ) and decisional aspects pertaining to how judgments are made and reported (the anchor points *S*_st_ or *S*_mp_, the observers' resolution δ and, where applicable, their response bias ξ). All of these components affect the psychometric function, including its location and slope. With reasonable assumptions about these components, Figures 4, 5 showed that neither the empirical location of the peak of Ψ_same_ and its gradient on either side nor the empirical location of the 50% point on Ψ_long_ and its slope can be interpreted as pure indices of timing processes. But the interpretation of data is further complicated if three other implicit assumptions of single-presentation methods are violated.

**Figure 6:**
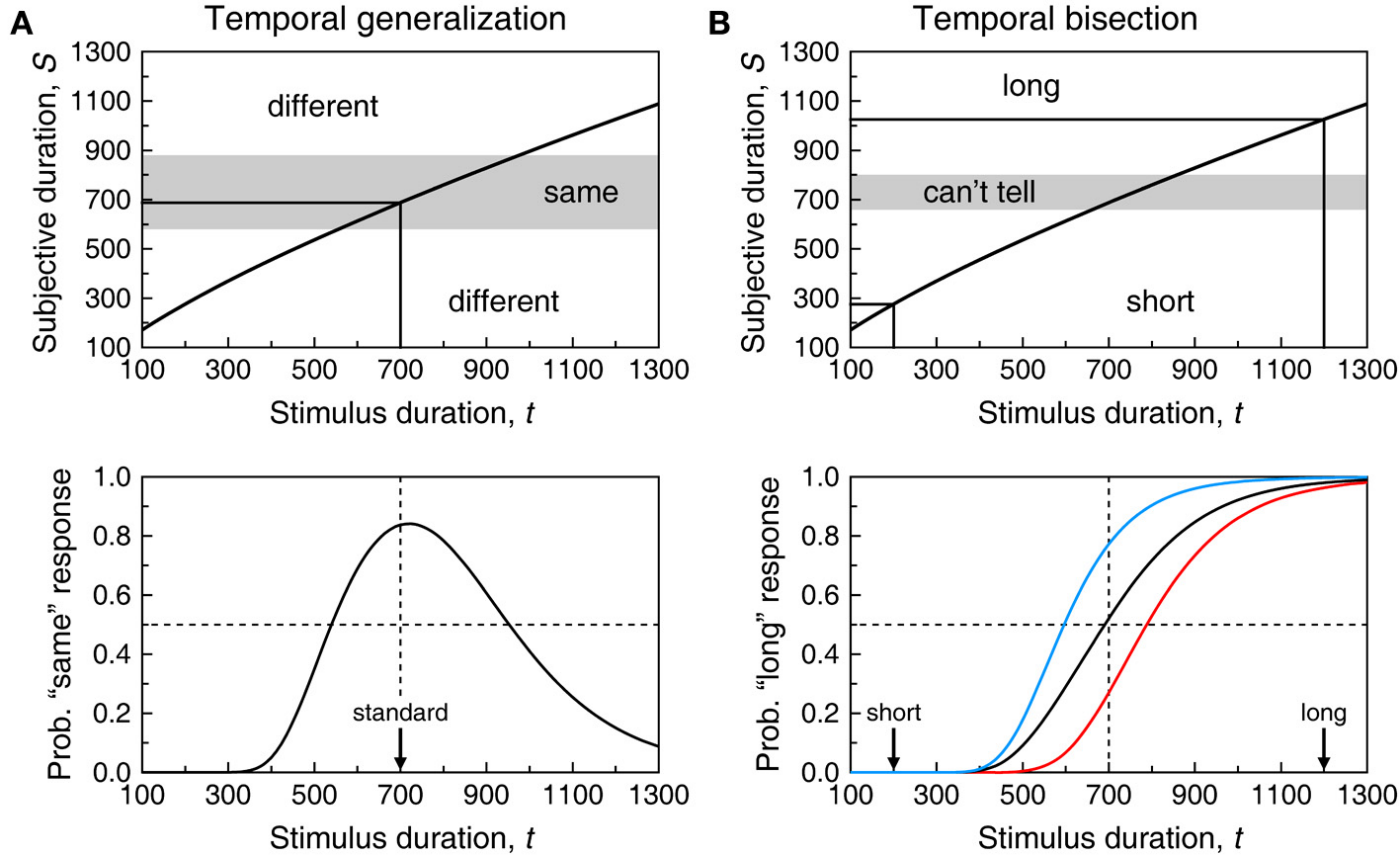
**Effects of decisional bias in the temporal generalization **(A)** and temporal bisection **(B)** tasks.** Graphical conventions as in Figures 4, 5. Decisional bias shows in that the central region for “same” or “I can't tell” judgments is displaced upwards by 30 units relative to its location in Figure 4 or 5. Psychometric functions are accordingly shifted laterally to the right.

The first assumption is that the indifference region is symmetric about the anchor point. In general, boundaries might be placed at *S*_st_ − δ_1_ and *S*_st_ + δ_2_ in the temporal generalization task (or at *S*_mp_ − δ_1_ and *S*_mp_ + δ_2_ in the bisection task), with symmetry occurring when δ_1_ = δ_2_ = δ. The effects of an asymmetric region are illustrated in Figure 6: Psychometric functions shift as a result of this *decisional bias*. Obtaining direct evidence of the symmetry of the indifference region is impossible with single-presentation data, but methods allowing this determination exist and their use has revealed that the indifference region is generally asymmetric (García-Pérez and Alcalá-Quintana, 2013; García-Pérez and Peli, 2014).

The second assumption is that the anchor points *S*_st_ and *S*_mp_ are respectively placed at μ_s_(*t*_st_) and at (μ_s_(*t*_short_) + μ_s_(*t*_long_))/2 during the training phase, as if observers used the arithmetic mean of a large sample of subjective durations elicited by repeated presentation of the standard (or the short and long exemplars). If the anchor were placed elsewhere during the training phase, the decision criterion during the test phase would not be at its presumed location and Ψ_same_ (or Ψ_long_) would shift accordingly. In these conditions, shifts of the psychometric function do not necessarily reflect differences in subjective time across training and test phases even if μ_s_(*t*) ≠ μ_*t*_(*t*) ≠ *t*. Obtaining evidence as to where the anchor point was placed seems impossible.

The third assumption is that anchors presumably placed at *S*_st_ = μ_s_(*t*_st_) or *S*_mp_ = (μ_s_(*t*_short_) + μ_s_(*t*_long_))/2 are stable. If they drifted systematically during the test phase, aggregating data across the session would shift the psychometric function. Concerns that anchor drift may occur come from adaptation level theory (Helson, 1948), which posits that the set of durations used during the test phase defines a context that relocates the internal standard. Stimulus range effects on temporal generalization do not seem to have been studied in a way that allows determining observable consequences on the location of Ψ_same_, but these effects have been reported for the bisection task (Wearden and Ferrara, 1995, 1996; Penney et al., 2014). The model of Wearden and Ferrara (1995) assumes that, as a result of this, the anchor is placed at the arithmetic mean of the set of test durations (or at 95% of this value; see Wearden, 2004). But the dynamics of the underlying processes are unknown, which precludes devising ways to eliminate or compensate for their effects so that bisection data are not contaminated by criterion placement.

These difficulties undermine the interpretation of temporal generalization and bisection data even under identical conditions in the training and test phases. Consider the bisection results reported by Gil et al. (2009). The training phase used a picture of an oval with *t*_short_ = 400 ms and *t*_long_ = 1600 ms so that *t*_mp_ = 1000 ms. Among conditions involving pictures of liked and disliked foods, the test phase also included a condition with the oval picture. Averaged across observers, results with the oval showed a remarkable shift: Ψ_long_ had its 50% point at *t* ≈ 800 ms, with Ψ_long_(*t*_mp_) ≈ 0.8. Assuming μ_s_ = μ_t_ = μ, σ_s_ = σ_t_ = σ, incorporating the scalar property, and removing the indifference region (i.e., δ = 0), Equation (5) reduces to Ψ_long_(*t*) = Φ (γ − *S*_mp_/σ(*t*)). If *S*_mp_ = μ(*t*_mp_) and τ in Equation (1) is removed, Ψ_long_(*t*) = Φ(γ − *t*^β^_mp_/γ*t*^β^) obtains. Reproducing the shape described by data from the oval condition in Gil et al.'s Figure 2 with this function requires β ≈ 2.39 and γ ≈ 1.46, unreasonable values compared to common estimates of β in μ and γ in the scalar property. Data are nonetheless unquestionable and a 50% point at *t* ≈ 800 ms with Ψ_long_(*t*_mp_) ≈ 0.8 are empirical facts. What is less clear is what the data say about the relation of subjective to objective time, or whether time is under- or over-estimated as opposed to veridically perceived. The same data could have arisen if β = 1 (i.e., μ(*t*) = *t*) and the assumption that *S*_mp_ = μ (*t*_mp_) is removed, implying that observers perceive duration veridically but for some reason they do not set the anchor at μ(*t*_mp_) during the training phase (Raslear, 1985; Allan and Gerhardt, 2001; Allan, 2002a,b). And the same shift could have been caused also with μ(*t*) = *t* and by reinstating the assumption that *S*_mp_ = μ (*t*_mp_) if observers had a non-null indifference region (i.e., δ ≠ 0) and responded with bias when undecided (Figure 5). Which scenario is responsible for the observed results is indiscernible because all account for the data equally well.

It is remarkable that virtually all analyses of bisection data have explicitly or implicitly assumed μ_t_(*t*) = *t* and, hence, that duration is accurately perceived. Yet, what should have thus been regarded as criterion shifts or response bias has been inconsistently interpreted as evidence of differences in perceived duration. To see that the assumption of veridical time perception is implicit when two-parameter psychometric functions are fitted to bisection data, make δ = 0 (i.e., a point criterion), μ_t_(*t*) = *t* (i.e., veridical time perception), and σ_t_(*t*) = *k* (i.e., remove the scalar property). In these conditions, Equation (5) becomes Ψ_long_(*t*) = Φ ((*t* − *S*_mp_)/*k*), which is the widespread cumulative Gaussian fitted to bisection data and sometimes replaced for convenience with a logistic function. On fitting this psychometric function to data, *S*_mp_ is regarded as a free parameter to account for observed shifts with respect to *t*_mp_, but this is synonymous with observers using an arbitrary criterion that varies across conditions (i.e., they do not set *S*_mp_ at *t*_mp_ in all conditions) and perceived duration being veridical and invariant across conditions (since μ_t_(*t*) = *t* in all cases).

Interpretation of bisection data is more difficult when the test phase does not include a condition with the training stimulus. Consider the results reported by Tipples (2010). Stimuli in the training phase were eight-consonant strings with *t*_short_ = 400 ms and *t*_long_ = 1600 ms so that *t*_mp_ = 1000 ms. The test phase used words of six different types: high arousal negative or positive, low arousal negative or positive, neutral, and sexual taboo. Since the 50% point on Ψ_long_ was 30–40 ms higher for taboo words than for the other types of word, Tipples concluded that time flies when one reads taboo words. Yet, and leaving other issues aside, without a reference provided by the 50% point on the psychometric function for eight-consonant strings, the 50% point on Ψ_long_ for test words is uninterpretable: The conclusion would have differed if the 50% point on Ψ_long_ for eight-consonant strings were above that for taboo words or below that for the other types of word. Tipple's conclusion is even more puzzling on consideration that, on average across observers, the 50% point lay between 955 and 970 ms for non-taboo words and nearly at 1000 ms for taboo words (see his Figure 2). Since *t*_mp_ = 1000 ms, the conventional (though unwarranted) conclusion should have been that time is perceived accurately only with taboo words.

These considerations apply also to the temporal generalization task, although studies assessing if time flies or slows down under certain conditions have almost exclusively used the bisection task. Measuring the psychometric function (be it Ψ_long_ or Ψ_same_) for training stimuli sets a reference for comparison with the psychometric function for other types of stimuli, but this does not solve the problems of single-presentation methods. The multiplicity of factors that can shift the psychometric function away from *t*_mp_ (or *t*_st_) preclude the interpretation of observed shifts as evidence of differences in subjective time across conditions. Bisection tasks are more seriously affected by this problem because response bias further alters the slope of the psychometric function (Figure 5) and contaminates DL estimates.

One might think that these problems would be solved by fitting psychometric functions such as those in Equations (3) or (5) to the data. Replacing the assumption of symmetry built into them (i.e., using δ_1_ and δ_2_ as needed instead of the single δ in them) puts into the fitted function all the factors that contribute to observed performance. Estimated parameter values would thus provide all the information needed for a proper interpretation of the data. With a psychophysical function given by Equation (1), estimates of the exponent β would directly indicate how time runs in each experimental condition provided the condition used to set the anchors is also tested. Unfortunately, models for single-presentation tasks are non-identifiable: There are infinite sets of parameter values that produce the same psychometric function (Yarrow et al., 2011; García-Pérez and Alcalá-Quintana, 2013; García-Pérez and Peli, 2014). This is not a problem of the models, but an indication that the intervening factors are inextricably confounded in single-presentation data. Data gathered with single-presentation methods are simply uninterpretable. Luckily, paired-comparison methods offer a suitable and dependable alternative with which these influences can be separated out.

## Paired-comparison methods

Trials in paired-comparison methods display two stimuli (a standard and a test, both of which may vary across trials) for observers to make a comparative judgment. Single-presentation methods imply a comparison too, but with respect to an internal standard. In paired-comparison tasks, the standard is explicit and subject to the same type of processing as is the test. A training phase is not needed to instate an internal standard, nor are assumptions about its placement and stability. Some modifications of the temporal generalization and bisection tasks turn them into paired-comparison methods, and the models discussed here apply to them too. For instance, the *roving standard* task of Allan and Gerhardt (2001) or Rodríguez-Gironés and Kacelnik (2001) presents in each trial a short and a long exemplar (which vary across trials) so that observers compare the test duration with the current exemplars. Similarly, the *episodic temporal generalization* task of Wearden and Bray (2001) presents a variable standard in each trial which is the reference for the observers' current judgment.

In paired-comparison trials, standard and test elicit subjective durations from the applicable distributions and observers judge by comparing the values drawn in the current trial. Observers can be asked to report whether both stimuli have the same subjective duration (the *equality task*), whether the first or the second appeared to have a longer duration (the *comparative task*), or whether the first, the second, or neither was subjectively longer than the other (the *ternary task*, which blends the two other tasks). [Incidentally, the bisection task can also be administered in a ternary format (Droit-Volet and Izaute, 2009) and its application reveals an indifference region whose width and symmetry differs across observers (García-Pérez and Peli, 2014).] The outcome of the timing component of a psychophysical model of performance in paired-comparison tasks cannot vary with the question asked at the end of the trial, as discussed in section A Unified Model of Performance across Semi-Objective Psychophysical Tasks. The next section describes the model for paired-comparison tasks, including a common timing outcome and a decision rule that varies with the task.

### The model for paired-comparison judgments

**Figure 7:**
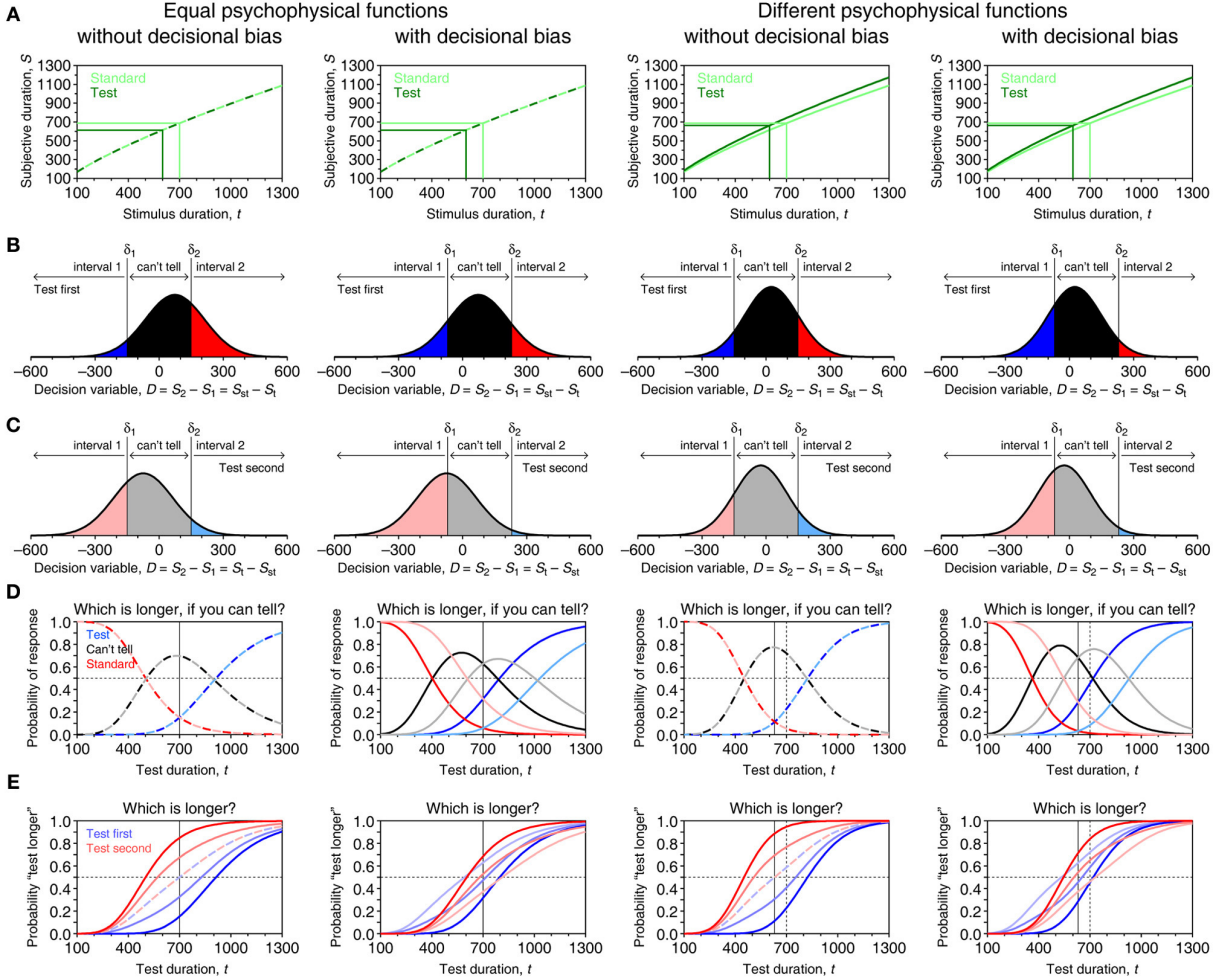
**Model-based psychometric functions for paired-comparison tasks in four different scenarios. (A)** Psychophysical functions μ_s_ (light green) and μ_t_ (dark green). In the two columns on the left, μ_s_ = μ_t_ with α_s_ = α_t_ = 5, β_s_ = β_t_ = 0.75, and τ_s_ = τ_t_ = −10 in Equation (1); in the two columns on the right μ_s_ is as before but parameters of μ_t_ are α_t_ = 5.4, β_t_ = 0.75, and τ_t_ = −10. Scalar variance is assumed with γ_s_ = 0.15 in all cases and γ_t_ = 0.15 also in the two left columns but γ_t_ = 0.10 in the two right columns. Light and dark green lines show in each scenario the mapping of the standard at *t*_st_ = 700 ms and a sample test at *t* = 600 ms. **(B)** Distribution of the decision variable for the standard-test pair just mentioned in a trial in which the test is presented first. The distribution is narrower in the two columns on the right due to the smaller variance. The decision space is partitioned into three regions by vertical lines at *D* = δ_1_ and *D* = δ_2_, with δ_1_ = −150 and δ_2_ = 150 in the first and third columns (i.e., no decisional bias) but δ_1_ = −70 and δ_2_ = 230 in the second and fourth columns (i.e., decisional bias). Each region is associated with the judgment indicated at the top, and the probability of the corresponding judgment equals the area under the distribution in that region. **(C)** Distributions in a trial involving the same pair but with the test presented in the second interval. **(D)** Psychometric functions in the ternary task for each presentation order. Color codes relate to **(B,C)** (e.g., dark blue denotes “interval 1” responses when the test was presented first; pale blue denotes “interval 2” responses when the test was presented second). A thin vertical line indicates the true location of the PSE defined as *t*_PSE_ = μ^−1^_t_ (μ_s_ (*t*_st_)). With parameters given above, *t*_PSE_ = 700 ms in the two columns on the left whereas *t*_PSE_ = 630.8 ms in the two columns on the right. **(E)** Psychometric functions for “test longer” responses in the comparative task for each presentation order and with response bias ranging from ξ = 0.5 (paler curves) through ξ = 0.75, to ξ = 1 (darker curves). The location of the true PSE is also indicated in each panel by a thin vertical line.

The model is analogous to an indecision model derived from SDT for use in other psychophysical tasks (García-Pérez and Alcalá-Quintana, 2010a,b, 2011a,b, 2013; Alcalá-Quintana and García-Pérez, 2011; García-Pérez and Peli, 2014). Its relation to other models will be discussed in section Differences with Previous Models. In the general case when standard and test differ qualitatively (as might be when test and standard are, e.g., eight-consonant strings vs. taboo words, or pictures of an oval vs. pictures of liked foods), the subjective duration *S*_st_ of a standard duration *t*_st_ is normally distributed with mean μ_s_(*t*_st_) and standard deviation σ_s_(*t*_st_) whereas the subjective duration *S*_t_ of a test duration *t* is normally distributed with mean μ_t_(*t*) and standard deviation σ_t_(*t*). Sample psychophysical functions that differ across test and standard stimuli are shown in the two right panels of Figure 7A. When standard and test stimuli are the same or when their differences do not affect subjective duration, μ_s_ = μ_t_ and σ_s_ = σ_t_ (two left panels of Figure 7A). Our goal here is to derive the psychometric function relative to a standard of fixed duration *t*_st_ across trials, whether or not such trials are interwoven with trials for other standards (which will define separate psychometric functions).

The model assumes that *S*_st_ and *S*_t_ are independent from one another and that observers' judgments are based on the magnitude of a decision variable *D* = *S*_2_ − *S*_1_ computed from the subjective durations of the second and first stimuli in the current trial. (The direction in which the difference is computed is immaterial, as will become evident below.) Because test and standard can (and should) be presented in either order with equal frequency across trials, each presentation order must be considered separately. Thus, on trials in which the test is presented first, *D* = *S*_st_ − *S*_t_ is normally distributed with mean μ_s_(*t*_st_) − μ_t_(*t*); on trials in which the test is presented second, *D* = *S*_t_ − *S*_st_ is also normally distributed but with mean μ_t_(*t*) − μ_s_(*t*_st_). In both cases the variance of *D* is the sum of the variances of *S*_st_ and *S*_t_. Figures 7B,C show the distributions of *D* for each presentation order on a trial with *t* = 600 ms when *t*_st_ = 700 ms, given the psychophysical functions in Figure 7A. Limited resolution also prevents observers from using a point criterion and the decision space is partitioned into three regions separated by boundaries δ_1_ and δ_2_, which are symmetric about *D* = 0 when δ_1_ = −δ_2_ (first and third panels in Figures 7B,C) and otherwise reflect a decisional bias (second and fourth panels in Figures 7B,C). Judgments turn into responses in a way that varies with the task.

Consider first a ternary task in which observers report whether duration was subjectively longer in the first interval, in the second, or in neither. Observers respond “interval 2” when *D* > δ_2_, “interval 1” when *D* < δ_1_, and “I can't tell” when δ_1_ ≤ D ≤ δ_2_ (see labels in the top part of Figures 7B,C). Response probabilities vary with presentation order due to the different mean of *D* in each case. Specifically, the probability Ψ_*i*_ of an “interval *i*” response when the test is presented in interval *i*, with *i* ∈ {1, 2}, varies with *t* as

(6a) $$\Psi_{1}(t)=\text{Prob}\left(S_{\text{st}}-S_{\text{t}}<\delta_{1}\right)=\Phi \text{​}\left(\frac{\delta_{1}-\mu_{\text{s}}\left(t_{\text{st}}\right)+\mu_{\text{t}}(t)}{\sqrt{\sigma_{\text{s}}^{2}\left(t_{\text{st}}\right)+\sigma_{\text{t}}^{2}(t)}}\right)$$

(6b) $$\Psi_{2}(t)=\text{Prob}\left(S_{\text{t}}-S_{\text{st}}>\delta_{2}\right)=1-\Phi \text{​}\left(\frac{\delta_{2}-\mu_{\text{t}}(t)+\mu_{\text{s}}\left(t_{\text{st}}\right)}{\sqrt{\sigma_{\text{s}}^{2}\left(t_{\text{st}}\right)+\sigma_{\text{t}}^{2}(t)}}\right)\text{​},\;\;\;\;\;\;\;\;$$

the probability ϒ_*i*_ of an “I can't tell” response when the test is presented in interval *i* varies with *t* as

(7a) $$\begin{array}{l} {ϒ}_{1}(t)=\text{Prob}\left(\delta_{1}\leq S_{\text{st}}-S_{t}\leq \delta_{2}\right)=\Phi \text{​}\left(\frac{\delta_{2}-\mu_{\text{s}}\left(t_{\text{st}}\right)+\mu_{\text{t}}(t)}{\sqrt{\sigma_{\text{s}}^{2}\left(t_{\text{st}}\right)+\sigma_{\text{t}}^{2}(t)}}\right)\\ \;-\Phi \text{​}\left(\frac{\delta_{1}-\mu_{\text{s}}\left(t_{\text{st}}\right)+\mu_{\text{t}}(t)}{\sqrt{\sigma_{\text{s}}^{2}\left(t_{\text{st}}\right)+\sigma_{\text{t}}^{2}(t)}}\right)\; \end{array}$$

(7b) $$\begin{array}{l} {ϒ}_{2}(t)=\text{Prob}\left(\delta_{1}\leq S_{\text{t}}-S_{\text{st}}\leq \delta_{2}\right)=\Phi \text{​}\left(\frac{\delta_{2}-\mu_{\text{t}}(t)+\mu_{\text{s}}\left(t_{\text{st}}\right)}{\sqrt{\sigma_{\text{s}}^{2}\left(t_{\text{st}}\right)+\sigma_{\text{t}}^{2}(t)}}\right)\\ \;-\Phi \text{​}\left(\frac{\delta_{1}-\mu_{\text{t}}(t)+\mu_{\text{s}}\left(t_{\text{st}}\right)}{\sqrt{\sigma_{\text{s}}^{2}\left(t_{\text{st}}\right)+\sigma_{\text{t}}^{2}(t)}}\right), \end{array}$$

and the probability of responding as the interval in which the standard was presented is 1 − Ψ_*i*_ − ϒ_*i*_. Figure 7D plots psychometric functions in each scenario and their features are discussed next.

The most conspicuous aspect is that psychometric functions do not differ across presentation orders in the absence of decisional bias (first and third columns in Figure 7) and they differ otherwise (second and fourth columns in Figure 7). Differences (or lack thereof) in the psychophysical functions for standard and test also have observable effects. Consider the PSE as a proxy to these differences. By definition, the PSE is the duration *t* that the test must have for its subjective duration to equal the subjective duration of the standard. Thus, the PSE is the duration *t*_PSE_ = μ^−1^_t_ (μ_s_ (*t*_st_)) and its location is readily identifiable in the psychometric functions. Consider the left column of Figure 7, where δ_1_ = −δ_2_ and *t*_PSE_ = *t*_st_ because μ_s_ = μ_t_. Here, Ψ_1_ (blue curve) crosses 1 − Ψ_2_ − ϒ_2_ (pale red curve) at *t* = *t*_st_ and Ψ_2_ (red curve) also crosses 1 − Ψ_1_ − ϒ_1_ (pale blue curve) at that point. It can be easily seen from Equations (6) to (7) that these crossings occur under any conditions at the duration *t* satisfying μ_t_(*t*) = μ_s_(*t*_st_). In contrast, ϒ_1_ and ϒ_2_ (black and gray curves) peak below *t* = *t*_st_ due to the scalar property. Hence, “I can't tell” responses are not maximally prevalent at the PSE and, thus, it is not the location of the peak of ϒ_1_ or ϒ_2_ that signals the PSE.

With decisional bias under the same conditions (second column in Figure 7), psychometric functions differ across presentation orders but the PSE is identically encoded because the crossing property holds always. In contrast to the preceding case, where ϒ_1_ and ϒ_2_ superimpose, their crossing here also occurs at the PSE. It can again be easily seen from Equations (7) that ϒ_1_(*t*) = ϒ_2_(*t*) at all *t* when δ_1_ = −δ_2_ (the conditions in the first column of Figure 7) and that they cross at the duration *t* satisfying μ_t_(*t*) = μ_s_(*t*_st_) when δ_1_ ≠ −δ_2_ (the conditions in the second column of Figure 7). The third and fourth columns in Figure 7 show that identification of the PSE is also not hampered when μ_s_ ≠ μ_t_ and σ_s_ ≠ σ_t_, as would occur when subjective time runs differently for test and standard. In the absence of decisional bias (third column), the crossing occurs at *t* = μ^−1^_t_ (μ_s_ (*t*_st_)) = 630.8 ms (thin vertical line); with decisional bias (fourth column), the crossings still occur at the same location. In sum, in the ternary paired-comparison task, the effects of decisional bias are not confounded with those of psychophysical functions that differ for standard and test. In this task, the “I can't tell” option also eliminates the contaminating influence of response bias because observers are not forced to give uninformative “interval 1” or “interval 2” responses when undecided.

The equality task, where observers report whether or not the two durations are subjectively equal, renders analogous outcomes. Observers respond “same” when they would have responded “I can't tell” in the ternary task whereas they respond “different” when they would have responded “interval 1” or “interval 2.” The psychometric functions in Equations (7) hold for the equality task, and the preceding discussion applies also to this task. It should be noted that the PSE is not identifiable by eye in ϒ_1_ and ϒ_2_ in the absence of decisional bias (i.e., when δ_1_ = −δ_2_ and the functions superimpose). This is not a problem, as will be discussed in section Summary and Discussion of Paired-Comparison Methods.

In contrast, the comparative task in which observers are forced to respond “interval 1” or “interval 2” calls again for a response bias parameter ξ describing how observers give arbitrary responses when they cannot tell which duration was longer. It should be clear by now that this can only bring complications. Assume that, as a result of response bias, observers respond “interval 2” with probability ξ when they cannot tell. In this task, “interval *i*” responses are translated as “test longer” when the test had been presented in interval *i*. The probability Ψ_*i*_ of “test longer” responses when the test was presented in interval *i* varies with *t* as

(8a) $$\begin{array}{l} \Psi_{1}(t)=\text{Prob​}\left(S_{\text{st}}-S_{\text{t}}<\delta_{1}\right)+\left(1-\xi \right)\text{Prob​}\left(\delta_{1}\leq S_{\text{st}}-S_{\text{t}}\leq \delta_{2}\right)\\ \;=\xi \text{​}\Phi \text{​​}\left(\frac{\delta_{1}-\mu_{\text{s}}\left(t_{\text{st}}\right)+\mu_{\text{t}}(t)}{\sqrt{\sigma_{\text{s}}^{2}\left(t_{\text{st}}\right)+\sigma_{\text{t}}^{2}(t)}}\right)\\ \;+\left(1\;-\;\xi \right)\text{​}\Phi \text{​​}\left(\frac{\delta_{2}-\mu_{\text{s}}\left(t_{\text{st}}\right)+\mu_{\text{t}}(t)}{\sqrt{\sigma_{\text{s}}^{2}\left(t_{\text{st}}\right)+\sigma_{\text{t}}^{2}(t)}}\right) \end{array}$$

(8b) $$\begin{array}{l} \Psi_{2}(t)=\text{Prob​}\left(S_{\text{t}}-S_{\text{st}}>\delta_{2}\right)+\xi \text{Prob​}\left(\delta_{1}\leq S_{\text{t}}-S_{\text{st}}\leq \delta_{2}\right)\\ \;=1-\xi \Phi \text{​}\left(\frac{\delta_{1}-\mu_{\text{t}}(t)+\mu_{\text{s}}\left(t_{\text{st}}\right)}{\sqrt{\sigma_{\text{s}}^{2}\left(t_{\text{st}}\right)+\sigma_{\text{t}}^{2}(t)}}\right)\\ \;-(1\;-\;\xi )\Phi \text{​}\left(\frac{\delta_{2}-\mu_{\text{t}}(t)+\mu_{\text{s}}\left(t_{\text{st}}\right)}{\sqrt{\sigma_{\text{s}}^{2}\left(t_{\text{st}}\right)+\sigma_{\text{t}}^{2}(t)}}\right). \end{array}$$

These psychometric functions are plotted in Figure 7E for sample values of ξ in each of the same four scenarios. The PSE is still defined with respect to the underlying psychophysical functions, but Figure 7D shows that the 50% point on the psychometric function does not relate to this definition. Consider again the first column of Figure 7, in which psychophysical functions are identical for test and standard and there is no decisional bias. The psychometric functions are identical for both presentation orders only when ξ = 0.5 (dashed curves) and their 50% point lies at the true PSE in such case; as ξ increasingly exceeds 0.5, Ψ_1_ (blue curves) shifts progressively to the right whereas Ψ_2_ (red curves) shifts progressively to the left, with their 50% points symmetrically placed with respect to the PSE. Both functions also turn progressively steeper in this transition and it is also clear that Ψ_1_ and Ψ_2_ have different shapes (i.e., they do not differ by translation only), which is another consequence of the scalar property. In the third column of Figure 7, still without decisional bias but when psychophysical functions differ for test and standard, the psychometric functions are displaced laterally toward the true PSE, maintaining the properties described above. Yet, with decisional bias (second and fourth columns), lack of response bias (ξ = 0.5) produces psychometric functions that also differ across presentation orders, although Ψ_1_ and Ψ_2_ still have their 50% points symmetrically placed around the true PSE.

Data from the comparative task are usually aggregated across presentation orders, although this practice is unadvisable (Ulrich and Vorberg, 2009). The resultant psychometric function is then Ψ_2AFC_(*t*) = (Ψ_1_(*t*) + Ψ_2_(*t*))/2 and it is easy to see from Equations (8) that the 50% point on Ψ_2AFC_ occurs at *t* = μ^−1^_t_ (μ_s_ (*t*_st_)). Thus, the average of the psychometric functions for each presentation order has its 50% point at the PSE, a result derived by Ulrich and Vorberg for the case in which μ_s_ = μ_t_ and generalized by García-Pérez and Alcalá-Quintana (2010a, 2011b) for the case in which μ_s_ ≠ μ_t_ and *t*_PSE_ ≠ *t*_st_. Although data from the comparative task are still useful for estimating the PSE, estimation of the true DL from percent points on the psychometric function is impossible due to the strong influence of response bias on its slope.

### Differences with previous models

Models for paired-comparison tasks are used in many areas of psychophysics. Almost all of them derive from SDT principles and share structural characteristics with our model, except that they do not include an indifference region (i.e., they assume δ_1_ = δ_2_), nor are they adapted to ternary tasks. With δ_1_ = δ_2_ = 0, Equations (8) become

(9) $$\Psi_{1}(t)=\Psi_{2}(t)=\Phi \text{​}\left(\frac{\mu_{\text{t}}(t)-\mu_{\text{s}}\left(t_{\text{st}}\right)}{\sqrt{\sigma_{\text{s}}^{2}\left(t_{\text{st}}\right)+\sigma_{\text{t}}^{2}(t)}}\right).$$

Such model does not seem to have been used in time perception. Conventional practice fits instead cumulative Gaussian or logistic functions to the data or, equivalently, fits straight lines to the *z*-scores of observed proportions. This entails a model analogous to (and with the same problems as) the model discussed in section Summary and Discussion of Single-Presentation Methods for bisection data. In the comparative task, the argument of the sigmoidal function is also of the form (*t* − *a*)/*b* and the consequences are identical: The free location parameter *a* replaces μ_s_(*t*_st_) in Equation (9) and allows the 50% point to be placed as needed without connection to the subjective duration of the standard; the free spread parameter *b* replaces the entire denominator of the argument of Φ in Equation (9), thus removing the scalar property; and replacement of μ_t_(*t*) with *t* amounts to assuming that subjective and objective time run identically. Succeeding in fitting such sigmoidal function and observing differences in estimated location parameters across conditions can only be justifiably interpreted as criterion shifts.

In contrast, a model proposed by Rammsayer and Ulrich (2001) does justice to the assumptions and goals of studies on time perception. In their model, consideration of the statistics of counting processes yielded a non-identity psychophysical function and subjective durations whose standard deviation increases with *t*. The model was also developed for application to the ternary task used to gather their empirical data. With appropriate replacements for μ and σ, their model and the resultant psychometric functions for the ternary task are identical to those in Equations (7) and (8) above except that Rammsayer and Ulrich set δ_1_ = −δ_2_ (i.e., no decisional bias). For unknown reasons, this model was subsequently abandoned by their proponents, as was the ternary task.

Another model for the comparative task was proposed by Dyjas et al. (2012). Again in comparison with our model, they assumed no indifference region (i.e., δ_1_ = δ_2_ = 0), undistorted time perception (i.e., μ_t_(*t*) = μ_s_(*t*) = *t*), non-scalar timing (i.e., σ_t_(*t*) = σ_s_(*t*) = σ, a constant), and a history component that alters an “internal standard” in line with adaptation level theory. The internal standard is updated on every trial as the convex sum of its value on the previous trial and the *subjective duration of the first interval* in the current trial. Such internal standards can be described as normally distributed with mean μ_s_(*t*_st_) = *t*_st_ and a standard deviation that varies with presentation order (see expressions for their variances in Equations 12–13 and 15–16 of Dyjas et al.). For simplicity, let σ_1_ and σ_2_ represent the equivalent standard deviation of the internal standard when the test is presented first or second. All of this turns Equations (8) into

(10) $$\Psi_{i}(t)=\Phi \text{​}\left(\frac{t-t_{\text{st}}}{\sqrt{\sigma_{i}^{2}+\sigma^{2}}}\right)\text{​},i\in \{1,2\}.$$

We use the term “equivalent” because substituting the expression for σ_1_ coming from Dyjas et al.'s (2012) Equation (16) into our Equation (10) does not render the psychometric function in their Equation (26). This is due to an additional term in the numerator of the argument in the first line of their Equation (26), which they transferred to the denominator in the second line. Also, the standard deviation of the internal standard varies according to whether the two presentation orders are blocked or randomly interwoven (see also Dyjas and Ulrich, 2014). The use of “equivalent” standard deviations permits our Equation (10) to cover all applicable cases while facilitating verbal descriptions of their model.

Participation of such internal standard was invoked to produce different slopes for Ψ_1_ and Ψ_2_, something that is accomplished by the different σ_*i*_ in Equation (10). The scalar property excluded from Dyjas et al.'s model would have produced the same effect (Figure 7E). Since Ψ_*i*_ in Equation (10) has its 50% point at *t* = *t*_st_ for all *i* while empirical data contradict this property, Dyjas et al. fitted their model using a logistic version of Equation (10) with *a*_*i*_ (in place of *t*_st_) and *b*_*i*_ (in place of $\sqrt{\sigma_{i}^{2}+\sigma^{2}}$) as free parameters subject to Ulrich and Vorberg's (2009) constraint. Since μ_t_(*t*) = μ_s_(*t*) = *t* is assumed, this implies that shifts of the psychometric function away from *t*_st_ are caused by criterion setting, not by differences in perceived duration. In a variant of this model, Dyjas and Ulrich (2014) displaced the point criterion to some arbitrary δ (i.e., δ_1_ = δ_2_ = δ), which turns Equation (10) into

(11) $$\Psi_{i}(t)=\Phi \text{​}\left(\frac{t-t_{\text{st}}-{\left(-1\right)}^{i}\delta }{\sqrt{\sigma_{i}^{2}+\sigma^{2}}}\right),i\in \{1,2\}.$$

The success of Equation (11) at fitting the empirical data of Dyjas and Ulrich provides further support to the notion that shifts of Ψ_1_ and Ψ_2_ can be attributed to criteria, not necessarily reflecting differences in perceived duration (which are explicitly excluded by their assumptions). Dyjas and Ulrich also presented a version of their model for the equality task, for which they introduced a potentially asymmetric indifference region. This renders psychometric functions identical to our Equations (7) with the amendments discussed above to include the participation of an internal standard. Their model for the equality task is thus incompatible with their model for the comparative task, as the latter assumes that observers never judge stimuli to have the same subjective duration. Interestingly, Dyjas et al. (2012) had allowed observers to hit a separate response key when they judged the two presentations in a trial to have the same duration, but they did not describe how those responses were treated and they presented and analyzed data as if such responses had never been given. Dyjas and Ulrich did not include this extra response option.

Dyjas and Ulrich also described a model including sensation weighting as implemented in the model of García-Pérez and Alcalá-Quintana (2011a), but this model is not discussed here because it is empirically indistinguishable from the internal standard model.

### Summary and discussion of paired-comparison methods

The shape of psychometric functions for paired-comparison tasks is determined by an embedded representation of subjective duration (μ and σ) and by aspects of the decision process. In contrast to single-presentation methods, paired-comparison methods are free of complications arising from untestable assumptions regarding the placement and stability of anchors. An added value of paired-comparison methods is that they lend themselves to a separate analysis of data for each presentation order (Figure 7), by which the influence of criteria and decisional bias on observed performance is separated from that of true differences in subjective duration (different μ for test and standard) or in its variance (different σ for test and standard).

But these are only potential benefits. If data are analyzed by fitting psychometric functions implying μ(*t*) = *t* in all cases, the potential of paired-comparison methods is wasted: Differences in observed performance across conditions can only be justifiably attributed to different criterion settings. To harvest the benefits, fitted psychometric functions must include a non-identity μ whose parameters capture the relation of subjective to objective time that best accounts for the data in each condition. The universally accepted scalar property should also be included in place of the fixed-variance assumption of typical analyses. Using subscripts for the parameters of μ and σ in Equations (1)–(2) (and setting τ = 0 for simplicity), Equations (6) and (7) for the ternary task become on substitution

(12a) $$\Psi_{1}(t)=\Phi \text{​}\left(\frac{\delta_{1}-\alpha_{\text{s}}t_{\text{st}}^{\beta_{\text{s}}}+\alpha_{\text{t}}t^{\beta_{\text{t}}}}{\sqrt{\gamma_{\text{s}}^{2}\alpha_{\text{s}}^{2}t_{\text{st}}^{\text{2}\beta_{\text{s}}}+\gamma_{\text{t}}^{2}\alpha_{\text{t}}^{2}t^{\text{2}\beta_{\text{t}}}}}\right)$$

(12b) $$\Psi_{2}(t)=1-\Phi \text{​}\left(\frac{\delta_{2}-\alpha_{\text{t}}t^{\beta_{\text{t}}}+\alpha_{\text{s}}t_{\text{st}}^{\beta_{\text{s}}}}{\sqrt{\gamma_{\text{s}}^{2}\alpha_{\text{s}}^{2}t_{\text{st}}^{\text{2}\beta_{\text{s}}}+\gamma_{\text{t}}^{2}\alpha_{\text{t}}^{2}t^{\text{2}\beta_{\text{t}}}}}\right)$$

(12c) $$\begin{array}{l} {ϒ}_{1}(t)=\Phi \text{​}\left(\frac{\delta_{2}-\alpha_{\text{s}}t_{\text{st}}^{\beta_{\text{s}}}+\alpha_{\text{t}}t^{\beta_{\text{t}}}}{\sqrt{\gamma_{\text{s}}^{2}\alpha_{\text{s}}^{2}t_{\text{st}}^{\text{2}\beta_{\text{s}}}+\gamma_{\text{t}}^{2}\alpha_{\text{t}}^{2}t^{\text{2}\beta_{\text{t}}}}}\right)\\ \;-\Phi \text{​}\left(\frac{\delta_{1}-\alpha_{\text{s}}t_{\text{st}}^{\beta_{\text{s}}}+\alpha_{\text{t}}t^{\beta_{\text{t}}}}{\sqrt{\gamma_{\text{s}}^{2}\alpha_{\text{s}}^{2}t_{\text{st}}^{\text{2}\beta_{\text{s}}}+\gamma_{\text{t}}^{2}\alpha_{\text{t}}^{2}t^{\text{2}\beta_{\text{t}}}}}\right) \end{array}$$

(12d) $$\begin{array}{l} {ϒ}_{2}(t)=\Phi \text{​}\left(\frac{\delta_{2}-\alpha_{\text{t}}t^{\beta_{\text{t}}}+\alpha_{\text{s}}t_{\text{st}}^{\beta_{\text{s}}}}{\sqrt{\gamma_{\text{s}}^{2}\alpha_{\text{s}}^{2}t_{\text{st}}^{\text{2}\beta_{\text{s}}}+\gamma_{\text{t}}^{2}\alpha_{\text{t}}^{2}t^{\text{2}\beta_{\text{t}}}}}\right)\\ \;-\Phi \text{​}\left(\frac{\delta_{1}-\alpha_{\text{t}}t^{\beta_{\text{t}}}+\alpha_{\text{s}}t_{\text{st}}^{\beta_{\text{s}}}}{\sqrt{\gamma_{\text{s}}^{2}\alpha_{\text{s}}^{2}t_{\text{st}}^{\text{2}\beta_{\text{s}}}+\gamma_{\text{t}}^{2}\alpha_{\text{t}}^{2}t^{\text{2}\beta_{\text{t}}}}}\right). \end{array}$$

Equations (12c)–(12d) apply also to the equality task, and a similar substitution in Equations (8) renders explicit functions for the comparative task. It should be noted from Figure 7E that response bias combined with a non-identity μ and the scalar property act together to produce the Type A and Type B order effects discussed by Ulrich and Vorberg (2009), which can thus be accounted for without ad hoc assumptions involving internal standards or sensation weighting.

Parameter estimates for these model-based psychometric functions can be easily obtained with maximum-likelihood methods. Technicalities are omitted here but empirical examples involving other classes of psychophysical functions are available (García-Pérez and Alcalá-Quintana, 2013; García-Pérez and Peli, 2014). Simulation studies have also shown that parameters can be recovered from data collected with the usual numbers of trials in empirical studies, but these results are too lengthy to be reported here.

It is also worth noting that performance measures such as PSEs or DLs can be computed from parameter estimates without reference to percent points on the psychometric functions. Indeed, since model parameters refer to underlying processes common to all tasks and not to aspects of the shape of the psychometric function for some task, PSEs and DLs can be computed according to their theoretical definition. As shown earlier, the PSE defined as *t*_PSE_ = μ^−1^_t_ (μ_s_ (*t*_st_)) can be obtained given the functional forms of μ_s_ and μ_t_ and estimates of their parameters. The DL, on the other hand, is usually computed as the distance between some percent points on the psychometric function for the comparative task. As seen in Figure 7E, the location of these points is greatly affected by the width and location of the indifference region and also by response bias. Ulrich and Vorberg (2009) proposed computing a separate DL from the psychometric function for each presentation order, but this practice also results in a description of time perception that is contaminated by all the non-timing processes that affect observed performance. Ultimately, computation of the DL seeks the durations satisfying, say, Prob(*S*_t_ > *S*_st_) = 0.25 and Prob(*S*_t_ > *S*_st_) = 0.75. Since parameter estimates give a full description of μ and σ for test and standard stimuli, uncontaminated estimates of the *latent* DL (García-Pérez and Alcalá-Quintana, 2012, 2013) can easily be obtained by noting that the latent point at which Prob(*S*_t_ > *S*_st_) = *p* is the duration *t*_*p*_ satisfying

(13) $$\Phi \text{​}\left(\frac{\alpha_{\text{t}}t_{p}^{\beta_{\text{t}}}+\alpha_{\text{s}}t_{\text{st}}^{\beta_{\text{s}}}}{\sqrt{\gamma_{\text{s}}^{2}\alpha_{\text{s}}^{2}t_{\text{st}}^{2\beta_{\text{s}}}+\gamma_{\text{t}}^{2}\alpha_{\text{t}}^{2}t_{p}^{2\beta_{\text{t}}}}}\right)=p,$$

an equation that can be directly solved from parameter estimates. DLs and WRs thus computed are free of the contaminants that affect the probability of observed responses, and they are also independent of the task with which the data were collected.

## General discussion and evidence-based recommendations

The pace of subjective time surely differs from that of objective time, and different stimulus types or conditions surely alter the pace of subjective time further. This means that the psychophysical function μ describing the relation of subjective to objective time cannot be the identity function and that its parameters must vary across conditions. Yet, studies in which semi-objective tasks have been used to assess differences in time perception routinely fit psychometric functions implying μ(*t*) = *t* in all conditions, also including a location parameter allowed to vary across conditions. If someone wanted to make the case that time perception is always accurate and different conditions only make observers set different response criteria, fitting such psychometric functions would be the way to gather supporting evidence. The success with which empirical data are accounted for with that type of psychometric function has nevertheless been taken as evidence of differences in perceived duration across conditions. Although the theoretical underpinnings of the fitted psychometric functions do not permit such interpretation, the overwhelming success with which data have historically been accounted for as if only criterion differences were involved cannot be taken as ruling out differences in subjective time across conditions. For a proper assessment of the various determinants of observed performance, model-based psychometric functions should be fitted to data to interpret the parameters describing each of the influences that affect performance. But data should also be collected using psychophysical tasks that allow separating out those influences. The following sections discuss what the theoretical analyses presented in this paper say about these issues.

### Psychophysical tasks

The model presented in this paper renders psychometric functions tailored to the characteristics of each semi-objective psychophysical task. The functions μ and σ describing subjective duration are always included in the psychometric functions and, in principle, the parameters of μ and σ could be estimated from data gathered with any task. But observed performance is also affected by decisional and response processes that lend additional parameters to the psychometric function, and not all psychophysical tasks provide informative data for an estimation of the parameters describing all of these influences. We showed that all determinants of performance are inextricably confounded in data gathered with single-presentation methods, which are thus unsuitable for assessing time perception (or any other perceptual process; see García-Pérez and Alcalá-Quintana, 2013). The use of single-presentation methods should be discontinued.

Paired-comparison methods, on the other hand, provide data from which these influences can be separated out, allowing a proper assessment of each of the determinants of performance. Of the various formats that paired-comparison methods may take, the ternary task is best suited for these purposes. It should be noted that any study conducted with a single-presentation method can also be conducted with the ternary paired-comparison task. Consider the studies of Gil et al. (2009) or Tipples (2010) discussed in section Summary and Discussion of Single-Presentation Methods, which used a bisection task to investigate whether subjective time runs differently for different types of stimuli. In a ternary paired-comparison task, each trial would present the standard (a picture of an oval or an eight-consonant string) with some fixed duration (say, *t*_st_ = 1000 ms) along with the test stimulus for a duration that varies across trials. Trials with different types of test stimuli (or different standard durations) could be randomly interwoven in a session and the order of presentation of test and standard in each trial would also be randomly determined. Fitting the psychometric functions in Equations (12) to the resultant data would thus provide estimates of the parameters of μ and σ that describe observed performance, permitting a proper assessment of how subjective time varies across conditions besides providing parameters describing decisional determinants. García-Pérez and Peli (2014) illustrated this approach in a study of spatial bisection that used the conventional single-presentation format and its conversion into the ternary paired-comparison format.

### Fitting psychometric functions

Current practice fits two-parameter (location and slope) psychometric functions separately to data from each of the conditions included in a study. Yet, when the same standard is used for all conditions, model parameters describing the perceived duration of the standard should not vary across them. Psychometric functions are thus expected to differ only in the parameters describing subjective duration for test conditions. This implies that psychometric functions ought to be fitted jointly across conditions with some of their parameters constrained to have common values across them. This strategy reduces the number of free parameters needed to describe the data but it also entails a coherent use of models and provides the means to test hypotheses concerning the effect of manipulations. There are several other situations in which some parameters must be regarded as common across conditions, but these are determined by the experimental design. For illustrative examples, see García-Pérez and Alcalá-Quintana (2007a; 2009a; 2012; under review), Magnotti et al. (2013), or García-Pérez and Peli (2014).

### Adaptive methods

**Figure 8:**
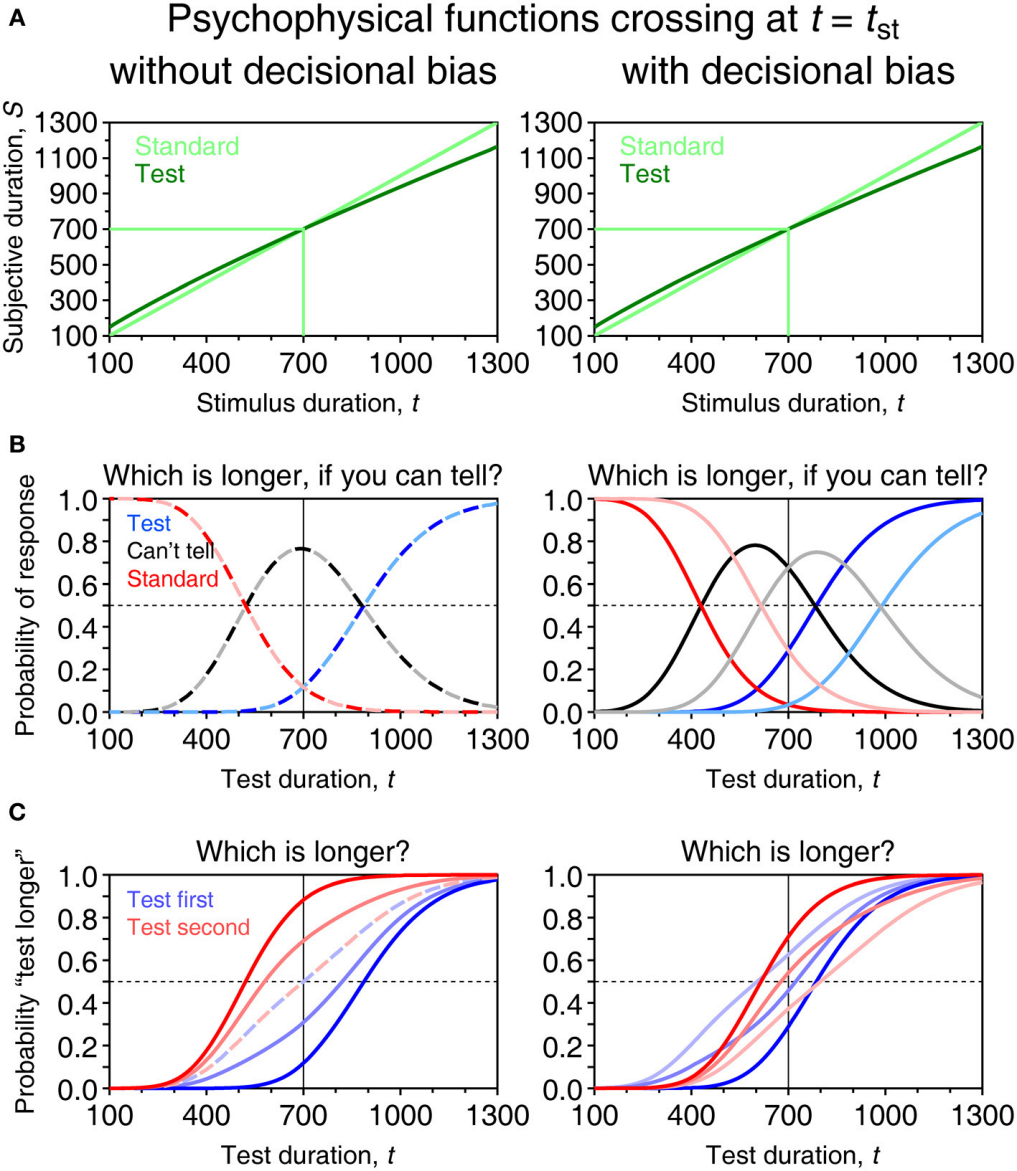
**The PSE at the standard duration is not a sufficient condition for equality of time perception for test and standard stimuli.** Graphical conventions and indifference regions for absence or presence of decisional bias as in Figure 7. **(A)** Different psychophysical functions for test and standard may cross at or very near *t* = *t*_st_. In this illustration, μ_s_ is the identity function whereas μ_t_ is given by Equation (1) with α_t_ = 3.01, β_t_ = 0.83, and τ_t_ = −10. As regards the scalar property in Equation (2), γ_s_ = 0.15 and γ_t_ = 0.10. **(B)** Psychometric functions in the ternary task, showing the signature of the PSE at *t* = *t*_st_ = 700 ms regardless of the presence (left column) or absence (right column) of decisional bias and despite the different psychophysical functions for test and standard. **(C)** Psychometric functions for “test longer” responses in the comparative task, again showing the effects of decisional and response bias.

Studies on time perception often use the comparative task to estimate PSEs or DLs via adaptive methods that bypass estimating the psychometric function, directly targeting specific percent points on it. This practice is unadvisable for several reasons. Firstly, and least importantly, μ_t_ may differ from μ_s_ in a way that they cross near *t* = *t*_st_. Thus, finding the PSE at or near *t*_st_ does not allow concluding that subjective duration is identical for test and standard stimuli (see Figure 8). Secondly, due to the effects of decisional and response bias on the slope and location of the psychometric function in comparative tasks, PSEs or DLs estimated from percent points are contaminated by these influences and do not portray time perception. Finally, and even in the absence of the previous two problems, the most widespread adaptive methods have been shown to provide percent-point estimates that are biased in magnitudes which cannot be assessed without knowledge of the shape of the psychometric function (García-Pérez, 1998, 2000, 2001, 2002, 2011; Alcalá-Quintana and García-Pérez, 2004, 2007; Faes et al., 2007; García-Pérez and Alcalá-Quintana, 2007b, 2009b; Hsu and Chin, 2014).

The foregoing discussion does not mean that adaptive methods should be entirely abandoned. On the contrary, some up–down methods provide dependable and efficient strategies for data collection and, thus, they gather maximally informative data for fitting psychometric functions. Adaptive methods tailored to the peculiarities of equality and ternary tasks have recently been developed (García-Pérez, 2014). For an illustration of their use, see García-Pérez and Peli (2014). What should be avoided by all means is the practice of estimating percent points by averaging reversal levels.

### Pending issues

It is unclear at this point whether the mathematical form of μ and σ in Equations (1)–(2) describe adequately the mean and standard deviation of subjective duration across the continuum from a few milliseconds to several seconds. Empirical studies suggest that a power function is adequate for μ within narrow time ranges but its parameters vary across ranges (Eisler, 1976), suggesting that a power function is only piecewise approximate. Although a yet unknown mathematical form might be more appropriate, the narrow range of durations used in any given study supports the use of Equation (1) on fitting psychometric functions. On the other hand, the scalar property in Equation (2) is known to be inaccurate in human timing but alternative mathematical forms have been proposed (Killeen et al., 1997; Rammsayer and Ulrich, 2001) that may prove more useful in practice. Also in this respect, it is unclear whether the referent for the scalar property is subjective time (as in Equation 2) or objective time.

Consideration of errors made by observers upon reporting judgments via the response interface has been intentionally excluded in this description. Extensions incorporating error parameters for more accurate parameter estimation have been discussed for analogous models elsewhere (García-Pérez and Alcalá-Quintana, 2012; García-Pérez and Peli, 2014) and their inclusion in the models presented here is straightforward.

### Conflict of interest statement

The author declares that the research was conducted in the absence of any commercial or financial relationships that could be construed as a potential conflict of interest.
